# Efficacy and tolerability of atypical antipsychotics for acute bipolar depression: a network meta-analysis

**DOI:** 10.1186/s12888-021-03220-3

**Published:** 2021-05-11

**Authors:** Aditi Kadakia, Carole Dembek, Vincent Heller, Rajpal Singh, Jennifer Uyei, Katsuhiko Hagi, Tadashi Nosaka, Antony Loebel

**Affiliations:** 1 Sunovion Pharmaceuticals Inc., Marlborough, MA USA; 2 IQVIA, Stockholm, Sweden; 3 IQVIA, Mumbai, India; 4 IQVIA, San Francisco, CA, USA; 5 Sumitomo Dainippon Pharma Co., Ltd., Tokyo, Japan

## Abstract

**Background:**

While clinical trial evidence has firmly established the efficacy of several atypical antipsychotics (AAPs) for treating bipolar depression, no randomized controlled trials (RCT’s) comparing AAPs have been conducted. This Bayesian network meta-analysis (NMA) compared the relative efficacy and tolerability of AAP monotherapy in adults with bipolar depression.

**Methods:**

Efficacy measures included change in Montgomery Åsberg Depression Rating Scale (MADRS), Clinical Global Improvement – Bipolar Disorder (CGI-BP), response, and remission. Multiple tolerability outcomes were examined. Results from random effects models were reported as difference in change from baseline for continuous variables or odds ratios for dichotomous variables. Treatments were ranked using the surface under the curve cumulative ranking probabilities. Number needed to treat (NNT) and harm (NNH) were calculated.

**Results:**

Eighteen RCT’s met inclusion criteria of the systematic literature review. On change in MADRS, lurasidone (− 4.71 [95% Crl − 6.98, − 2.41]), quetiapine (− 4.80 [− 5.93, − 3.72]), olanzapine (− 4.57 [− 5.92, − 3.20]), and cariprazine (− 2.29 [− 3.47, − 1.09]) were more efficacious than placebo. Lurasidone was associated with a significantly greater odds of response (≥50% improvement in MADRS) compared to cariprazine (1.78 [95% Crl 1.08, 2.77]), aripiprazole (2.38 [1.38, 3.85]), and ziprasidone (2.47 [1.41, 3.98]), but was similar to olanzapine (1.68 [0.99,2.65]) and quetiapine (1.25 [0.78, 1.90]). For change in CGI-BP-S-overall score, lurasidone was significantly better than cariprazine (− 0.38 [95% Crl − 0.66,-0.10]) and ziprasidone (− 0.58 [− 0.91,-0.26]), but similar to quetiapine (− 0.08 [− 0.36, 0.19])and olanzapine (− 0.04 [− 1.41, 1.46]). Lurasidone (0.34 kg [95% Crl − 0.22, 0.89]) and aripiprazole (0.20 kg [− 0.59, 1.00]) had a similar weight change compared to placebo, but olanzapine (2.88 kg [2.40, 3.36]), quetiapine (1.17 kg [0.84, 1.49]), and cariprazine (0.65 kg [0.34, 0.96]) were associated with greater weight gain. The NNT for response was the lowest for lurasidone (NNT = 5) followed by quetiapine (NNT = 6), olanzapine (NNT = 10) and cariprazine (NNT = 12).

**Conclusions:**

In this NMA in adults with bipolar depression, which evaluated change in depressive symptoms (assessed by MADRS) across short-term trials, the largest improvement versus placebo was observed for lurasidone, olanzapine and quetiapine with cariprazine, showing a smaller treatment effect. Aripiprazole and ziprasidone were ineffective for the treatment of bipolar depression. Improvement in CGI-BP-S score for lurasidone was larger than cariprazine and ziprasidone but similar to quetiapine and olanzapine. Based on short term studies lurasidone and aripiprazole had similar weight gain compared to placebo.

**Supplementary Information:**

The online version contains supplementary material available at 10.1186/s12888-021-03220-3.

## Background

Bipolar disorder is a chronic mood disorder characterized by episodes of mania (or hypomania), depression, and periods with normal mood (euthymia) [1]. Two forms of bipolar disorder are bipolar I disorder, which requires a history of at least one manic episode, and bipolar II disorder, which requires a history of at least 1 hypomanic episode and one major depressive episode [1]. The estimated yearly and lifetime prevalence for bipolar I- II disorders is 2.8 and 4.4% respectively [2, 3]. In 2015, the cost of bipolar I disorder in the United States was estimated at $81,559 per patient, made up predominately of unemployment (36%), caregiving (25%), and direct healthcare (23%) costs [4].

In bipolar I disorder, depression is the predominant abnormal mood state [5], with depressive symptoms being 3 times more common than manic symptoms [6]. Bipolar depression, but not bipolar mania, is associated with increased rates of unemployment [7] and among those who are employed, bipolar depression is associated with more absenteeism, presenteeism, and total lost workdays than bipolar mania [7, 8]. In addition suicide risk is substantially higher during depressive episodes than manic episodes [9].

The recommended pharmacological treatments for bipolar disorder vary depending on the phase of the disorder (acute depression, acute mania, or maintenance). Pharmacological treatments used for acute bipolar depression come from multiple different classes including atypical antipsychotics, anticonvulsants, lithium, and antidepressants.

The US Food and Drug Administration (FDA) has approved four medications, all atypical antipsychotics, for the treatment of bipolar depression: quetiapine, lurasidone, cariprazine, and the combination of olanzapine with fluoxetine. Lurasidone is the only available atypical antipsychotic indicated for treatment of bipolar depression in combination with lithium or valproate. Lurasidone, as monotherapy and in combination with lithium or valproate, and quetiapine are recommended as first-line treatments for the management of acute bipolar depression in the 2018 Canadian Network for Mood and Anxiety Treatment (CANMAT) and International Society for Bipolar Disorders (ISBD) guidelines. The CANMAT/ISBD guidelines recommend cariprazine and olanzapine-fluoxetine for second-line treatment. The use of antidepressants to treat bipolar depression remains controversial due to limited evidence for efficacy and the potential risk of switching into mania or inducing rapid cycling [10, 11].

Several meta-analyses [12–14] and network meta-analyses (NMAs) of atypical antipsychotics in bipolar depression have been conducted [15, 16]. NMAs allow for comparisons to be made between drugs that have a common comparator, such as placebo, even though no head-to-head trials have been conducted. While the methods and study inclusion criteria have varied, the meta-analyses have consistently reported that quetiapine, olanzapine, and lurasidone are more efficacious than placebo. Other atypical antipsychotics monotherapies have not consistently shown superior efficacy compared to placebo [12–16]. Only one prior NMA evaluated tolerability outcomes and found differences among the atypical antipsychotics [15]. Lurasidone was associated with significantly less weight gain than quetiapine and olanzapine and significantly less somnolence than quetiapine and ziprasidone. There were no significant differences observed in the rates of all-cause discontinuation between lurasidone and other atypical antipsychotics [15].

Since these NMAs were conducted, additional clinical trials of atypical antipsychotic monotherapies have been completed. The objective of this study was to update a recent NMA [15] to better understand the relative efficacy, safety and tolerability of currently available atypical antipsychotics approved for the treatment of bipolar depression.

## Methods

### Systematic literature review

A systematic literature review was conducted to identify randomized controlled trials of atypical antipsychotic monotherapy in bipolar depression. A protocol was developed for this review and NMA and can be found in Additional file 1, however it was not registered. The amendments to the protocol are listed in Additional file 1 section of protocol. The PRISMA 2020 expanded statement for reporting of systematic reviews incorporating network meta-analyses can be found in the Additional file 1: Table 1 [17]. The most recent bipolar depression NMA identified trials that were completed prior to May 2015 [15]. This update included searches in Embase, MEDLINE, Cochrane Library, and PsycINFO for studies published between May 2015 and 04 May 2020. Eligibility criteria for trial inclusion remained consistent with the previous NMA [15], which was developed using the Patient, Intervention, Comparator, Outcome, and Study type (PICOS) paradigm to minimize bias and identify as many relevant studies as possible [18]. To be included in this NMA, studies must have been double-blinded randomized controlled trials (RCTs) of adults (≥18 years old), with either bipolar I disorder or bipolar II disorder (at least 50% with bipolar I disorder), treated with an atypical antipsychotic as monotherapy, and reported at least one outcome of interest at study endpoint of week 8 or less (Table 1). The exact search terms and resulting number of records returned are reported in Additional file 1: Table 2a. In addition, conference abstracts were reviewed for the 2019–2020 meetings of 11 psychiatry professional organizations to identify secondary publications to supplement already included studies that were published in peer reviewed journals (Additional file 1: Table 2b). A clinical trial registry (https://www.clinicaltrials.gov/) was searched for additional studies on 04 May 2020 and during the data extraction process. The registry was searched using keywords related to the interventions listed in the PICOS and disease terms related to bipolar I disorder. Study investigators, authors and companies were not contacted for additional data and only the published data was used for this review.

**Table 1:** Study Inclusion and Exclusion Criteria

Criterion	Inclusion	Exclusion
**Patient population**	Adults (> 18 years old) with bipolar depression where at least 50% of the population were diagnosed with bipolar I disorder	•< 50% bipolar I disorder •< 18 years old
**Interventions**	Atypical antipsychotic monotherapy: •Cariprazine^1^ •Lurasidone •Quetiapine •Olanzapine •Aripiprazole •Asenapine •Risperidone •Ziprasidone •Brexpiprazole^1^ •Lumateperone^1^ •All other atypical antipsychotic monotherapies assessed for the treatment of bipolar I depression^2^	•Any treatments other than those listed in the inclusion criteria •Any treatments listed in inclusion criteria if administered as adjunctive therapy
**Comparators**	Any of the above listed interventions or placebo	Comparators other than those listed in the inclusion criteria
**Outcomes**	Studies reporting at least one of the following outcomes: •Change from baseline in MADRS •Change from baseline in CGI-BP-S •Response (defined as ≥50% improvement inMADRS) •Remission (defined as MADRS score ≤ 12 and ≤ 10at endpoint) •≥ 7% weight gain •Change from baseline in weight •Change from baseline in glucose level •Change from baseline in low-density lipoprotein (LDL) •Change from baseline in total cholesterol •Change from baseline in triglycerides •Change from baseline in prolactin •Akathisia •Extrapyramidal symptoms •Somnolence •All-cause discontinuation •Discontinuation due to lack of efficacy •Discontinuation due to adverse events •Switch to mania^3^	Studies not reporting least one of the outcomes included in the inclusion criteria.
**Study design**	Randomized controlled trials	•Non-randomized controlled trials •Observational studies •Case studies •Pharmacology studies

*Abbreviations*: *CGI-BP-S* Clinical Global Impressions – Bipolar Disorder – Severity, *MADRS* Montgomery–Åsberg Depression Rating Scale. ^1^treatments were added to the update since they were approved after the original search in 2015. ^2^Other atypical antipsychotic monotherapies were allowed in the update to ensure any new treatments were not excluded. ^3^Switch to mania was added post-hoc

### Study selection

All references/publications were screened based on title and abstract against the inclusion and exclusion criteria by two independent reviewers and discrepancies were resolved by a third reviewer. The full text of publications retained during abstract review were again reviewed by two independent reviewers and similarly discrepancies resolved by a third reviewer. Following best practices, data extraction from selected studies was conducted by two independent researchers with results cross-checked to ensure accuracy.

### Outcome variables

The primary efficacy outcome was change from baseline in the MADRS total score reported week 8 or before. In addition, change in CGI-BP-S-overall and CGI-BP-S-depression scores, response rate (≥ 50% improvement from baseline in MADRS), and remission rate (MADRS ≤12 and ≤ 10 at endpoint) were also examined. Discontinuation outcomes included all-cause discontinuation, discontinuation due to adverse events, and discontinuations due to lack of efficacy. Metabolic outcomes included change in weight, rate of ≥7% weight gain, changes in triglycerides, total cholesterol, low-density lipoprotein (LDL) cholesterol, glucose, and prolactin. Additional tolerability outcomes included rates of investigator reported somnolence, extrapyramidal symptoms (EPS), akathisia, and switch to mania.

Missing standard errors (SEs) for continuous outcomes were estimated from reported standard deviations, 95% confidence intervals, *p* values or standard errors (reported for baseline and endpoint values) [18].

### Network meta-analysis methods

The NMA was conducted according to guidance published by National Institute for Health and Care Excellence’s Decision Support Unit [19] and the International Society of Pharmacoeconomics and Outcomes Research Task Force on Indirect Treatment Comparisons [20]. When available, results from mixed models for repeated measures (MMRM) were favored over those using the last observational carried forward method to handle missing data. For trials with multiple fixed dose arms, the results were pooled across dose, which is consistent with methods used in several past meta-analyses [15, 16]. Network diagrams were drawn for each outcome and can be found in Additional file 1: Figure 1a-d.

The NMA was conducted with a Bayesian framework using OpenBUGS v3.2.3 (OpenBUGS Foundation) and R 3.6.1 (R Foundation for Statistical Computing, Vienna, Austria), following codes provided in the NICE Decision Support Unit (DSU) Technical Support Document 2 (TSD2) [21]. The methodology also followed guidance from the ISPOR Task Force on Indirect Treatment Comparisons [20, 22]. In addition, correlations induced by multi-arm trials were taken into account using the methods and codes recommended in the DSU TSD2 [21]. Continuous variables were modeled assuming an identity link and a normal distribution. Dichotomous variables were modeled using a logit link and binomial distribution. Results for continuous variables were reported as the difference in change from baseline and dichotomous variables were reported as odds ratios (OR). All results were reported along with the 95% credible intervals, the Bayesian analogue to confidence intervals. Treatments were ranked using the surface under the curve cumulative ranking (SUCRA) probabilities [23]. This ranking hierarchy was obtained by ordering the effects from the most to least effective (or tolerable) treatment in comparison to placebo. The base case models were fit with random effects models, which estimate additional variance parameters associated with study heterogeneity and generally have larger credible intervals than fixed effects models.

### Number needed to treat or harm

The number needed to treat, and number needed to harm were computed using risk differences derived from the network meta-analysis results following methods described in the Cochrane Handbook for Systematic Reviews of Interventions Version 6.1.0 [18, 24]. NNT and NNH values were calculated by taking the reciprocal of the absolute values of the risk difference [25, 26]. NNT and NNH were rounded up to the next whole number. NNT is a measure of effect size that indicates how many patients would need to be treated with the medication of interest instead of a comparator (i.e., placebo in the case of all trials included in the present study) for a single patient to benefit. Lower NNT values represent superior performance of the treatment of interest on a given outcome. NNH is similar to NNT but measures the number of patients who would likely need to be administered a treatment in order for a single patient to encounter the adverse event. Higher values for NNH represent better performance (i.e., a greater number of patients are likely to be treated before a single patient experiences an adverse event).

### Sensitivity analyses

We examined the impact of pooled vs. stratified doses of each atypical antipsychotics as well as restricted the data at the 6-week timepoint (i.e., data reported at other timepoints was removed.

### Quality of evidence and heterogeneity

The Grading of Recommendation, Assessment, Development and Evaluation (GRADE) approach for NMA was used to evaluate the confidence of evidence [27, 28]. The evidence was evaluated for each direct comparison within each network separately, and since there were no closed loops, an evaluation of indirect comparison was not applicable. Ratings were based on the following domains: study design, risk of bias, inconsistency, indirectness, imprecision, and publication bias [28]. Risk of bias was assessed using the Cochrane Risk of Bias version 2 (RoB2) [24, 29]. Publication bias was assessed by comparison-adjusted funnel plots, with tests for asymmetry applied to cases with > = 10 studies [30].

The transitivity assumption of NMA was evaluated by comparing the distribution of potential effect modifies across clinical trials that were included in the NMA, to ensure that their populations were suitably comparable. The following baseline patient characteristics were examined: age, gender, body weight, BMI, percent with bipolar I disorder, baseline MADRS, baseline CGI-BP-S depression score, and age of bipolar onset. In the NMA random effects models, a common heterogeneity parameter across the various treatment comparisons was assumed, and heterogeneity was assessed by the between-study variance τ^2^ (tau squared) for each outcome, and further characterized by comparing with its predictive distribution [31]. Since there were no closed loops in the NMA, assessment of incoherence was not applicable to this analysis.

## Results

### Literature review update

**Fig. 1:**
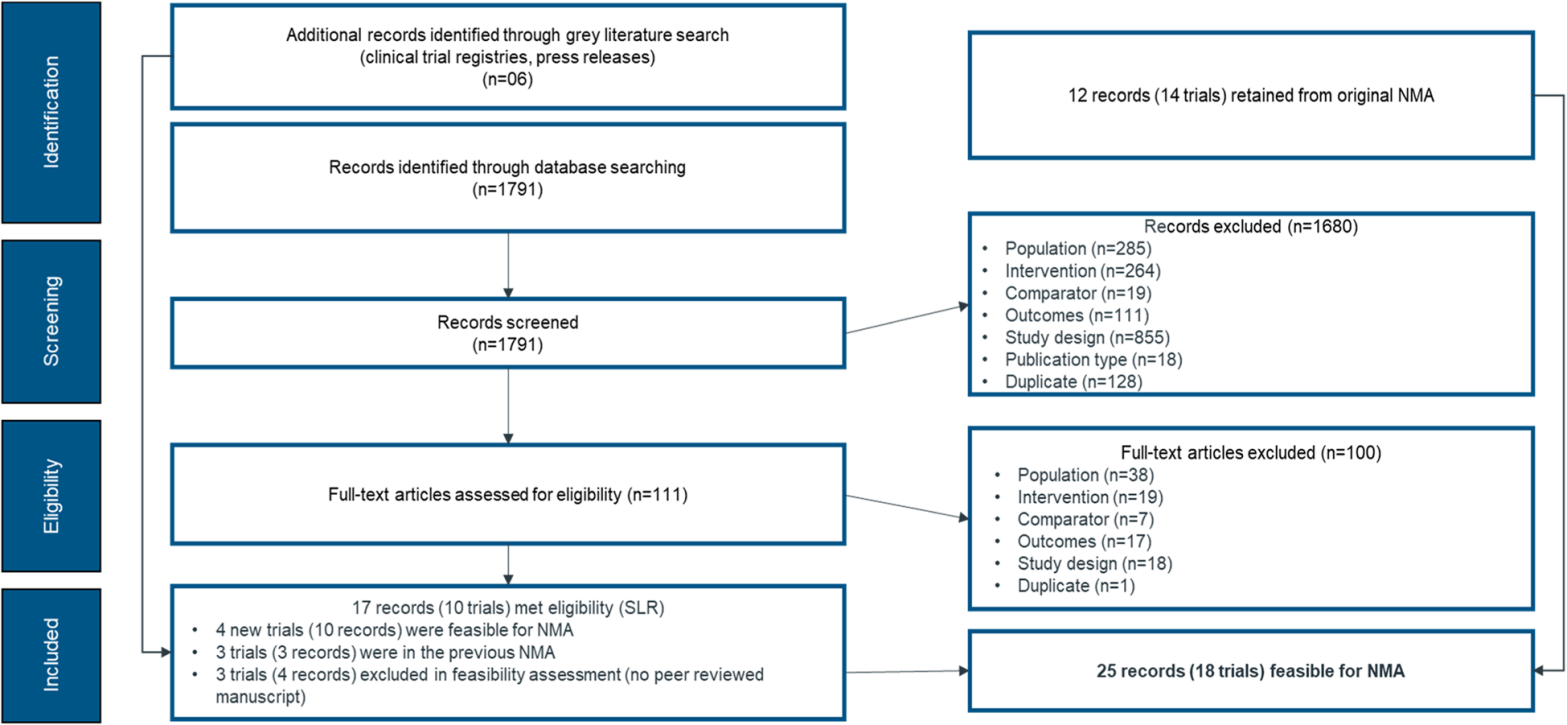
PRISMA flow diagram

The update to the systematic literature review identified 1791 records in EMBASE, MEDLINE, the Cochrane Library, and PsycINFO for screening (Fig. 1). A total of 111 full-text articles and secondary conference abstracts were reviewed with 17 records meeting all the inclusion criteria. In addition, 6 more records were identified from searches of the conference abstracts. These 17 records reflected 10 unique RCTs, 4 of which [32–35] had not been included in the earlier NMA [15]. Additionally, the results from one RCT that had been included in the earlier NMA based on a conference presentation has since been published [36]. Study results which were published only in conference abstracts and not in a peer-reviewed manuscript were excluded. A total of 18 trials from 25 references were included in the NMA [32–47]. Figure 1 gives the PRISMA flow diagram showing the reasons for record exclusion.

### Study characteristics

All studies were multi-site, randomized, double-blind, and placebo-controlled clinical trials. There were no head-to-head trials of atypical antipsychotics. Most trials were multi-national, but eight recruited only from sites in the United States (US) [35, 37, 39, 41–43] and two recruited only from sites in the People’s Republic of China [36, 46]. Most trials lasted 8 weeks in duration, but six studies [33, 34, 38, 39, 44] lasted only 6 weeks in duration.

Table 2 gives the baseline characteristics used in the assessment of heterogeneity for each of the included RCTs. The study populations were similar in terms of mean age (29.2–43.6 years), gender (34.3–48.1% male) distribution, mean baseline MADRS score (26.9–32.0), and mean baseline CGI-BP-S-overall (4.2–4.5) and depression (4.3–4.9) scores. In the quetiapine trials [36, 37, 40, 41, 43, 47], as few as 50.9% of patients were diagnosed with bipolar I disorder (the remainder were diagnosed with bipolar II disorder), whereas in the other trials 100% of patients were diagnosed with bipolar I disorder. Mean baseline body weight was not reported across all studies, but in 11 studies [32–34, 36, 38, 40–42, 46, 47] where it was reported, baseline body weight ranged from 63.9 to 88.8 kg. Mean age of onset was only reported in three trials [32, 38, 44], and ranged from 25.4 to 28.4 years.

**Table 2:** Study Design and Patient Baseline Characteristics

				Baseline Characteristics						
Study	n	Duration	Treatments	Age	Male	Weight^**a**^	Bipolar I	MADRS	CG-BP-S-Overall	CGI-BP-S- Depression
Calabrese et al.	511	8 weeks	Quetiapine IR 300 mg Quetiapine IR 600 mg Placebo	37.4	41.9%	NR	66.9%	30.4	4.4	NR
Durgam et al.	571	8 weeks	Cariprazine 0.75 mg Cariprazine 1.5 mg Cariprazine 3.0 mg Placebo	41.9	37.7%	80.9	100.0%	30.6	4.4	NR
Earley et al. Study 1)	480	6 weeks	Cariprazine 1.5 mg Cariprazine 3.0 mg Placebo	42.8	40.8%	86.5	100.0%	30.6	4.5	NR
Earley et al. Study 2)	490	6 weeks	Cariprazine 1.5 mg Cariprazine 3.0 mg Placebo	43.6	37.3%	84.78	100.0%	31.4	4.5	NR
Li et al.	279	8 weeks	Quetiapine XR 300 mg Placebo	33.1	48.1%	64.5	50.9%	28.7	NR	4.5
Loebel et al.^b^	485	6 weeks	Lurasidone 20–60 mg Lurasidone 80–120 mg Placebo	41.5	41.4%	77.2	100.0%	30.5	4.5	4.5
Lombardo et al. Study 1)	504	6 weeks	Ziprasidone 40–80 mg Ziprasidone 120–160 mg Placebo	39.9	43.6%	NR	100.0%	28.2	NR	NR
Lombardo et al. Study 2)	381	6 weeks	Ziprasidone 20–80 mg Placebo	40.2	42.5%	NR	100.0%	28.4	NR	NR
McElroy et al.	582	8 weeks	Quetiapine IR 300 mg Quetiapine IR 600 mg Placebo	38.5	36.9%	80.8	64.0%	26.9	4.2	NR
Suppes et al.	270	8 weeks	Quetiapine XR 300 mg Placebo	39.5	35.5%	88.8	80.4%	30.0	4.5	NR
Thase et al.	467	8 weeks	Quetiapine IR 300 mg Quetiapine IR 600 mg Placebo	37.7	43.1%	NR	67.4%	30.2	4.5	NR
Thase et al. (Study 1)	374	8 weeks	Aripiprazole 5–30 mg Placebo	39.0	37.5%	87.2	100.0%	28.8	NR	4.3
Thase et al. (Study 2)	375	8 weeks	Aripiprazole 5–30 mg Placebo	40.5	40.0%	86.8	100.0%	29.0	NR	4.4
Tohen et al.	747	8 weeks	Olanzapine > 5 mg^c^ Placebo	42.0	41.5%	NR	100.0%	32.0	NR	4.9
Tohen et al.	514	6 weeks	Olanzapine 10–20 mg Placebo	35.5	44.3%	NR	100.0%	29.0	NR	4.5
Wang et al.	68	8 weeks	Olanzapine 10–20 mg Placebo	29.2	41.2%	63.9	100.0%	28.6	4.4	NR
Young et al.	647	8 weeks	Quetiapine IR 300 mg Quetiapine IR 600 mg Placebo	42.2	41.6%	75.5	61.6%	28.3	4.3	NR
Yatham et al.	224	8 weeks	Cariprazine 0.25–0.75 mg Cariprazine 1.5–3.0 mg Placebo	38.9	34.3%	NR	72.7%	30.4	4.4	NR

*Abbreviations*: *NR* Not reported, *IR* Immediate release, *XR* Extended release

^a^ Weight is reported in kilograms (kg)

^b^ Data not reported in the publication by Loebel et al., 2014 was supplemented by data from the Clinical Study Report for lurasidone (Sunovion Pharmaceuticals, data on file)

^c^ Mean daily dose: 9.7 mg

^d^ All dosing is reported in mg/day

### Efficacy measures

Lurasidone, olanzapine, quetiapine, and cariprazine (but not aripiprazole or ziprasidone) were all significantly more efficacious than placebo in change from baseline in MADRS but there was a larger magnitude of change for lurasidone, quetiapine and olanzapine versus placebo (Table 3). According to SUCRA rankings, lurasidone, olanzapine and quetiapine ranked first for change in MADRS score versus placebo followed by cariprazine, ziprasidone and aripiprazole (Table 14). In pairwise comparison for change from baseline in MADRS, lurasidone was similar to cariprazine, olanzapine, and quetiapine, and was significantly better than aripiprazole and ziprasidone (Table 3).

**Table 3:** Change from Baseline in MADRS and Odds Ratio for Response (≥ 50% improvement in MADRS)

**PLO**	−1.08 [−3.26, 1.10]	**−2.29 [−3.47, −1.09]**	**−4.57 [−5.92, −3.16]**	**−4.80 [−5.93, − 3.72]**	− 1.34 [− 3.29, 0.67]	**−4.71 [−6.98, − 2.41]**
1.11 [0.80, 1.51]	**ARI**	− 1.21 [− 3.70, 1.29]	**−3.49 [− 6.07, −0.92]**	**−3.72 [− 6.21, − 1.28]**	−0.27 [− 3.24, 2.71]	**−3.63 [− 6.78, − 0.50]**
**1.47 [1.17, 1.82]**	1.35 [0.90, 1.95]	**CAR**	**−2.29 [− 4.09, − 0.46]**	**−2.52 [− 4.11, − 0.92]**	0.94 [− 1.34, 3.27]	−2.42 [− 5.01, 0.14]
**1.56 [1.20, 2.01]**	1.44 [0.94, 2.14]	1.08 [0.76, 1.51]	**OLA**	−0.23 [−2.05, 1.53]	**3.23 [0.83, 5.66]**	− 0.13 [− 2.82, 2.50]
**2.09 [1.74, 2.49]**	**1.92 [1.32, 2.71]**	**1.44 [1.08, 1.91]**	1.36 [0.98, 1.84]	**QUE**	**3.46 [1.24, 5.76]**	0.10 [−2.44, 2.61]
1.07 [0.77, 1.46]	0.99 [0.61, 1.50]	0.74 [0.50, 1.08]	0.70 [0.45, 1.03]	**0.52 [0.35, 0.74]**	**ZIP**	**−3.36 [− 6.38, − 0.39]**
**2.58 [1.67, 3.81]**	**2.38 [1.38, 3.85]**	**1.78 [1.08, 2.77]**	1.68 [0.99, 2.65]	1.25 [0.78, 1.90]	**2.47 [1.41, 3.98]**	**LUR**

Note: MADRS results are on the top-right and response results are on the bottom-left. Results give the mean change and odds ratio [95% credible interval]. In the top-right comparisons, the row treatment is the reference category. In the bottom-left, the column treatment is the reference category. Response was defined as ≥50% improvement in MADRS

*Abbreviations*: *MADRS* Montgomery–Åsberg Depression Rating Scale, *PLO* Placebo, *ARI* Aripiprazole, *CAR* Cariprazine, *LUR* Lurasidone, *OLA* Olanzapine, *QUE* Quetiapine, *ZIP* Ziprasidone

For response (≥50% improvement in MADRS score), lurasidone, quetiapine, olanzapine and cariprazine (but not aripiprazole and ziprasidone) were associated with significantly greater odds of response compared to placebo (Table 3). According to SUCRA rankings lurasidone ranked the first in terms of response followed by quetiapine, olanzapine, cariprazine, aripiprazole and ziprasidone as compared to placebo (Table 14). In pairwise comparison for response lurasidone had significantly greater odds of response than cariprazine, aripiprazole, and ziprasidone (Table 3).

For change in CGI-BP-S-overall score, lurasidone, cariprazine and quetiapine were significantly better than placebo, but olanzapine and ziprasidone were not. According to SUCRA rankings lurasidone ranked first in improving the overall CGI-BP-S score followed by quetiapine, olanzapine, cariprazine and ziprasidone (Table 14). In pairwise comparison for change in CGI-BP-S-overall score, lurasidone was associated with a significantly larger improvement than cariprazine and ziprasidone but showed similar improvements to quetiapine and olanzapine. Quetiapine was significantly better than cariprazine and ziprasidone in improving the overall severity assessed by CGI-BP-S (Table 4).

**Table 4:** Change from Baseline in CGI-BP-S-depression and CGI-BP-S-overall

**PLO**	NA	**−0.25 [− 0.38,-0.11]**	−0.59 [− 2.06, 0.76]	**−0.55 [− 0.68–0.41]**	−0.05 [− 0.26, 0.17]	**−0.63 [− 0.87,-0.39]**
−0.21 [− 0.63, 0.20]	**ARI**	NA	NA	NA	NA	NA
NA	NA	**CAR**	−0.34 [− 1.82, 1.01]	**−0.30 [− 0.49,-0.11]**	0.20 [− 0.05, 0.46]	**−0.38 [− 0.66,-0.10]**
−0.32 [− 0.72, 0.08]	−0.11 [− 0.68, 0.47]	NA	**OLA**	0.04 [− 1.31, 1.51]	0.54 [− 0.83, 2.03]	−0.04 [− 1.41, 1.46]
NA	NA	NA	NA	**QUE**	**0.50 [0.24, 0.76]**	−0.08 [− 0.36, 0.19]
NA	NA	NA	NA	NA	**ZIP**	**−0.58 [− 0.91,-0.26]**
**− 0.63 [− 1.12,-0.14]**	−0.42 [− 1.06, 0.23]	NA	− 0.31 [− 0.95, 0.33]	NA	NA	**LUR**

Note: CGI-BP-S-overall are on the top-right and CGI-BP-S-depression results are on the bottom-left. Numbers represent the mean change [95% credible interval]. In the top-right comparisons, the row treatment is the reference category. In the bottom-left, the column treatment is the reference category

*Abbreviations*: *CGI-BP-S* Clinical Global Impressions–Bipolar–Severity Scale, *PLO* Placebo, *ARI* Aripiprazole, *CAR* Cariprazine, *LUR* Lurasidone, *OLA* Olanzapine, *QUE* Quetiapine, *ZIP* Ziprasidone

For studies reporting on the change in CGI-BP-S-depression score, lurasidone was significantly better than placebo, but olanzapine and aripiprazole were not (Table 4). No studies reporting on CGI-BP-S-depression score were identified for cariprazine, quetiapine, and ziprasidone. Lurasidone ranked first in improving the CGI-BP-S-depression score followed by olanzapine and aripiprazole (Table 14).

The odds of remission (MADRS ≤12) were significantly greater for lurasidone, quetiapine, and olanzapine compared to placebo, but not ziprasidone (Table 5). The most effective treatment in improving remission rates as per the SUCRA rankings was lurasidone, followed by quetiapine, olanzapine ad ziprasidone (Table 14). In pairwise comparison for remission, lurasidone had greater odds of remission compared to ziprasidone and quetiapine had greater odds of remission compared to olanzapine as well as ziprasidone. Clinical trials for cariprazine defined remission as MADRS ≤10 at endpoint, and data for this definition was available only for lurasidone. Both lurasidone and cariprazine had significantly greater odds of remission compared to placebo (Table 5); and lurasidone ranked higher than cariprazine according to SUCRA rankings (Table 14).

**Table 5:** Odds Ratios for Remission as MADRS ≤12 and Remission as MADRS ≤10

**PLO**	**1.61 [1.12,2.23]**	NA	NA	NA	**2.16 [1.14, 3.76]**
NA	**CAR**	NA	NA	NA	1.39 [0.66, 2.60]
**1.44 [1.10, 1.88]**	NA	**OLA**	NA	NA	NA
**2.04 [1.70, 2.44]**	NA	**1.45 [1.02, 1.99]**	**QUE**	NA	NA
1.03 [0.75, 1.39]	NA	0.73 [0.47, 1.06]	**0.51 [0.35, 0.72]**	**ZIP**	NA
**2.20 [1.35, 3.40]**	NA	1.56 [0.86, 2.53]	1.09 [0.64, 1.73]	**2.19 [1.21, 3.64]**	**LUR**

Note: Remission defined as MADRS ≤10 results are on the top-right and Remission defined as MADRS ≤12 results are on the bottom-left. Numbers represent the odds ratio [95% credible interval]. In the top-right comparisons, the row treatment is the reference category. In the bottom-left, the column treatment is the reference category. Studies that used alternative definitions were not included: the aripiprazole studies and one quetiapine study defined remission as MADRS ≤8 at endpoint

*Abbreviations*: *PLO* Placebo, *CAR* Cariprazine, *LUR* Lurasidone, *OLA* Olanzapine, *QUE* Quetiapine, *ZIP* Ziprasidone

### Discontinuation rates

All-cause discontinuation for lurasidone, olanzapine, cariprazine and, ziprasidone, were comparable to placebo. However, the odds of all-cause discontinuation for aripiprazole were significantly higher than placebo (Table 6). Based on SUCRA ranking, olanzapine and quetiapine ranked the best tolerated treatments in terms of all-cause discontinuation followed by cariprazine, lurasidone, ziprasidone and aripiprazole (Table 14). For discontinuations due to adverse events, lurasidone, cariprazine, olanzapine and ziprasidone had similar odds compared to placebo. Aripiprazole and quetiapine had significantly higher odds of discontinuation due to adverse events compared to placebo. According to SUCRA ranking lurasidone and olanzapine ranked first in terms of discontinuation due to adverse events followed by cariprazine, ziprasidone, aripiprazole and quetiapine. In pairwise comparison, for discontinuations due to adverse events there were no significant differences between the atypical antipsychotics (Table 6). For discontinuation due to lack of efficacy, quetiapine and olanzapine had a significantly lower odds of discontinuation than placebo (Table 7). Based on SUCRA values, quetiapine ranked first in terms of discontinuation due to a lack of efficacy followed by olanzapine, cariprazine, lurasidone, aripiprazole and ziprasidone (Table 14). No significant differences were found for discontinuation due to lack of efficacy between placebo, and lurasidone, cariprazine, aripiprazole, and ziprasidone (Table 7).

**Table 6:** Odds Ratios for All-Cause Discontinuation and Discontinuation Due to Adverse Events

**PLO**	**1.68 [1.09, 2.48]**	1.05 [0.77, 1.41]	0.70 [0.48, 1.00]	0.99 [0.78, 1.24]	1.38 [0.92, 1.98]	1.10 [0.61, 1.83]
**2.47 [1.10, 4.90]**	**ARI**	0.66 [0.38, 1.06]	**0.44 [0.24, 0.73]**	**0.62 [0.37, 0.96]**	0.86 [0.47, 1.45]	0.68 [0.32, 1.28]
1.50 [0.82, 2.64]	0.70 [0.25, 1.64]	**CAR**	0.68 [0.42, 1.06]	0.96 [0.64, 1.37]	1.34 [0.79, 2.11]	1.07 [0.54, 1.90]
1.43 [0.68, 2.58]	0.67 [0.21, 1.57]	1.04 [0.37, 2.23]	**OLA**	1.46 [0.93, 2.18]	**2.03 [1.15, 3.30]**	1.62 [0.79, 2.95]
**2.46 [1.57, 3.75]**	1.15 [0.44, 2.49]	1.79 [0.80, 3.42]	1.93 [0.82, 4.09]	**QUE**	1.41 [0.88, 2.15]	1.12 [0.59, 1.95]
1.54 [0.76, 2.80]	0.72 [0.23, 1.70]	1.12 [0.41, 2.37]	1.21 [0.43, 2.82]	0.66 [0.27, 1.32]	**ZIP**	0.83 [0.40, 1.54]
1.12 [0.36, 2.76]	0.52 [0.12, 1.50]	0.81 [0.21, 2.23]	0.88 [0.22, 2.50]	0.48 [0.14, 1.24]	0.81 [0.20, 2.27]	**LUR**

Note: All-Cause Discontinuation results are on the top-right and Discontinuation Due to Adverse Events are in the bottom left. Results give the odds ratio [95% credible interval]. In the top-right comparisons, the row treatment is the reference category. In the bottom-left, the column treatment is the reference category

*Abbreviations*: *PLO* Placebo, *ARI* Aripiprazole, *CAR* Cariprazine, *LUR* Lurasidone, *OLA* Olanzapine, *QUE* Quetiapine, *ZIP* Ziprasidone

**Table 7:** Odds Ratios for Discontinuation Due to Lack of Efficacy

**PLO**	0.65 [0.19, 1.60]	0.50 [0.20, 1.04]	**0.42 [0.14, 0.93]**	**0.23 [0.12, 0.40]**	1.84 [0.54, 4.88]	0.77 [0.18, 2.17]
	**ARI**	1.02 [0.21, 3.06]	0.86 [0.16, 2.68]	0.47 [0.11, 1.30]	3.79 [0.62, 13.01]	1.58 [0.21, 5.73]
		**CAR**	1.01 [0.23, 2.83]	0.54 [0.17, 1.33]	4.41 [0.89, 14.22]	1.84 [0.31, 6.17]
			**OLA**	0.68 [0.19, 1.84]	**5.55 [1.03, 19.22]**	2.32 [0.35, 8.31]
				**QUE**	**8.91 [2.12, 26.64]**	3.72 [0.71, 11.61]
					**ZIP**	0.57 [0.07, 2.06]
						**LUR**

Note: All-Cause Discontinuation results are on the top-right and Discontinuation Due to Adverse Events results are on the bottom-left. Numbers represent the odds ratio [95% credible interval]. In the top-right comparisons, the row treatment is the reference treatment. In the bottom-left, the column treatment is the reference category

*Abbreviations*: *PLO* Placebo, *ARI* Aripiprazole, *CAR* Cariprazine, *LUR* Lurasidone, *OLA* Olanzapine, *QUE* Quetiapine, *ZIP* Ziprasidone

### Metabolic parameters measures

All atypical antipsychotics except lurasidone and aripiprazole were associated with significantly more weight gain than placebo. During the short-term trials, olanzapine had the largest mean weight gain relative to placebo (2.88 kg), followed by quetiapine (1.17 kg), cariprazine (0.65 kg), lurasidone (0.34 kg), and aripiprazole (0.20 kg). Olanzapine had significantly greater weight gain than all other antipsychotics and quetiapine had significantly greater weight gain than lurasidone and aripiprazole (Table 8). The SUCRA rankings further confirm that aripiprazole and lurasidone were most likely to be associated with smaller changes in weight from baseline followed by cariprazine, quetiapine and olanzapine (Table 14). All atypical antipsychotics except lurasidone and aripiprazole were associated with significantly greater odds of 7% weight gain versus placebo. In pairwise comparison for clinically significant weight gain (≥7%) olanzapine had significantly greater odds of weight gain compared to quetiapine, cariprazine and aripiprazole (Table 8). Descriptively, the rates of clinically significant weight gain were the lowest for lurasidone (2.4%) followed by cariprazine (3.2%), aripiprazole (4.7%)), quetiapine (6.9%)), and olanzapine (20.3%).

**Table 8:** Change from Baseline in Weight (kg) and Odds Ratios of ≥7% Weight Gain

**PLO**	0.20 [−0.59, 1.00]	**0.65 [0.34, 0.96]**	**2.88 [2.40, 3.36]**	**1.17 [0.84, 1.49]**	0.34 [− 0.22, 0.89]
1.67 [0.56, 3.92]	**ARI**	0.44 [− 0.42, 1.30]	**2.68 [1.76, 3.61]**	**0.96 [0.10, 1.83]**	0.14 [−0.85, 1.11]
**3.50 [1.26, 8.65]**	2.67 [0.56, 8.41]	**CAR**	**2.24 [1.66, 2.80]**	**0.52 [0.07, 0.96]**	−0.31 [− 0.95, 0.33]
**68.46 [15.56, 231.00]**	**52.56 [7.42, 205.90]**	**24.93 [3.35, 95.57]**	**OLA**	**−1.71 [−2.29, −1.13]**	**−2.54 [−3.28, − 1.81]**
**3.46 [1.91, 5.92]**	2.64 [0.74, 6.90]	1.26 [0.34, 3.13]	**0.08 [0.01, 0.25]**	**QUE**	**−0.83 [− 1.48, − 0.18]**
19.08 [0.66, 108.10]	14.68 [0.35, 83.24]	6.85 [0.16, 39.89]	0.43 [0.01, 2.56]	5.98 [0.18, 33.08]	**LUR**

Note: Weight Change results are on the top-right and numbers represent the mean change [95% credible interval]. ≥7% Weight Gain results are on the bottom-left and results give the odds ratio [95% credible interval]. In the top-right comparisons, the row treatment is the reference category. In the bottom-left, the column treatment is the reference category

*Abbreviations*: *PLO* Placebo, *ARI* Aripiprazole, *CAR* Cariprazine, *LUR* Lurasidone, *OLA* Olanzapine, *QUE* Quetiapine

There were no significant differences for change in total cholesterol among the atypical antipsychotics, but olanzapine was associated with greater increases in total cholesterol than placebo (Table 9). There were no significant differences from placebo or between atypical antipsychotics for change in triglycerides (Table 9), LDL (Table 10) or glucose (Table 10), during these acute trials.

**Table 9:** Change from Baseline in Triglycerides and Total Cholesterol

**PLO**	0.98 [− 10.48, 12.47]	1.35 [− 1.27, 6.50]	1.85 [− 1.88, 8.64]	11.10 [− 2.75, 24.86]	− 3.05 [− 15.37, 9.55]
0.50 [−5.64, 6.60]	**ARI**	0.37 [− 11.45, 12.83]	0.87 [− 11.26, 13.80]	10.12 [−7.63, 28.42]	−4.03 [− 21.02, 12.83]
− 2.05 [− 5.90, 1.67]	− 2.55 [−9.87, 4.56]	**CAR**	0.50 [− 5.46, 6.84]	9.75 [− 4.84, 24.02]	− 4.40 [− 17.54, 8.47]
**7.06 [2.47, 12.00]**	6.55 [− 1.05, 14.47]	9.11 [3.22, 15.46]	**OLA**	9.25 [− 5.75, 23.54]	− 4.89 [− 18.60, 8.37]
0.50 [− 4.86, 5.88]	0.00 [−8.13, 8.18]	2.55 [− 3.96, 9.20]	−6.55 [− 13.85, 0.46]	**QUE**	− 14.15 [− 32.40, 4.08]
1.72 [−6.56, 9.94]	1.22 [− 9.02, 11.51]	3.77 [−5.24, 12.88]	− 5.34 [− 14.99, 4.03]	1.21 [− 8.64, 10.98]	**LUR**

Note: Triglycerides results are on the top-right and Total Cholesterol results are on the bottom-left. Numbers represent the mean change [95% credible interval]. In the top-right comparisons, the row treatment is the reference category. In the bottom-left, the column treatment is the reference category

*Abbreviations*: *PLO* Placebo, *ARI* Aripiprazole, *CAR* Cariprazine, *LUR* Lurasidone, *OLA* Olanzapine, *QUE* Quetiapine

**Table 10:** Change from Baseline in Low-Density Lipoprotein Cholesterol and Glucose

**PLO**	− 0.50 [− 4.09, 3.14]	−0.67 [− 3.23, 0.42]	0.42 [− 1.23, 2.16]	−0.59 [− 4.23, 3.00]	1.18 [− 3.86, 6.23]
0.90 [− 2.17, 4.12]	**ARI**	− 0.17 [− 4.61, 3.60]	0.92 [− 3.03, 4.83]	−0.09 [− 5.29, 4.95]	1.67 [− 4.52, 7.81]
0.07 [− 1.31, 1.70]	− 0.83 [− 4.29, 2.59]	**CAR**	1.09 [− 0.48, 4.43]	0.08 [− 3.80, 4.44]	1.84 [− 3.28, 7.39]
−0.34 [− 3.18, 2.17]	−1.25 [− 5.62, 2.71]	−0.42 [− 3.78, 2.39]	**OLA**	−1.01 [− 5.01, 2.96]	0.75 [− 4.51, 6.04]
1.15 [− 0.82, 3.12]	0.25 [− 3.56, 3.91]	1.08 [− 1.46, 3.44]	1.50 [− 1.72, 4.94]	**QUE**	1.76 [− 4.42, 7.84]
−1.45 [−5.50, 2.64]	− 2.35 [− 7.61, 2.73]	−1.52 [− 5.85, 2.77]	−1.11 [− 5.85, 3.94]	−2.60 [− 7.12, 1.92]	**LUR**

Note: Low-Density Lipoprotein results are on the top-right and Glucose results are on the bottom-left. Numbers represent the mean change [95% credible interval]. In the top-right comparisons, the row treatment is the reference category. In the bottom-left, the column treatment is the reference category

*Abbreviations*: *PLO* Placebo, *ARI* Aripiprazole, *CAR* Cariprazine, *LUR* Lurasidone, *OLA* Olanzapine, *QUE* Quetiapine

### Other tolerability measures

Lurasidone was associated with higher changes in prolactin than placebo, aripiprazole, and quetiapine; and cariprazine was also associated with higher changes in prolactin than placebo (Table 11). According to SUCRA ranking for changes in prolactin aripiprazole ranked first followed by quetiapine, cariprazine and lurasidone (Table 14). All antipsychotics except lurasidone, cariprazine, and aripiprazole had greater somnolence than placebo, with quetiapine having greater somnolence than all antipsychotics except ziprasidone (Table 12). On SUCRA analysis, lurasidone ranked the best tolerated option in terms of somnolence followed by cariprazine, aripiprazole, olanzapine, quetiapine and ziprasidone (Table 14). Switch to mania for all antipsychotics was comparable to placebo, except for quetiapine which had a significantly lower odds of switching. Quetiapine also had lower odds of switch to mania compared with aripiprazole (Table 12) and ranked the best according to SUCRA values (Table 14). Rates of EPS were higher than placebo for lurasidone, quetiapine, and cariprazine, but there were no significant differences among the atypical antipsychotics (Table 13). Aripiprazole and cariprazine ranked the best tolerated options in terms of EPS followed by quetiapine and ziprasidone (Table 14). Among the atypical antipsychotics where data on akathisia was reported (aripiprazole, quetiapine, and lurasidone), odds were higher than placebo (Table 13). According to SUCRA rankings, cariprazine and lurasidone ranked the best tolerated options with fewer akathisia rates followed by aripiprazole (Table 14).

**Table 11:** Change from Baseline in Prolactin

**PLO**				
0.37 [− 1.72, 2.42]	**ARI**			
**2.22 [0.89, 3.57]**	1.85 [− 0.60, 4.35]	**CAR**		
1.04 [−1.42, 3.55]	0.67 [−2.57, 3.91]	−1.18 [− 4.02, 1.70]	**QUE**	
**7.20 [2.06, 12.33]**	**6.83 [1.25, 12.37]**	4.98 [− 0.36, 10.34]	**6.16 [0.41, 11.79]**	**LUR**

Note: Prolactin results are on the bottom-left. Numbers represent the mean change from baseline [95% credible interval]. In the top-right comparisons, the row treatment is the reference category. In the bottom-left, the column treatment is the reference category

*Abbreviations*: *PLO* Placebo, *ARI* Aripiprazole, *CAR* Cariprazine, *LUR* Lurasidone, *QUE* Quetiapine

**Table 12:** Odds Ratios for Somnolence and Switch to Mania

**PLO**	2.04 [0.98, 3.79]	1.90 [0.81, 4.05]	**2.89 [1.96, 4.20]**	**4.90 [3.59, 6.56]**	**5.05 [2.61, 9.25]**	1.53 [0.57, 3.66]
2.25 [0.81, 5.19]	**ARI**	1.04 [0.32, 2.61]	1.59 [0.68, 3.16]	**2.70 [1.21, 5.31]**	2.79 [0.98, 6.44]	0.84 [0.24, 2.28]
0.91 [0.41, 1.71]	0.50 [0.13, 1.33]	**CAR**	1.79 [0.64, 3.85]	**3.03 [1.13, 6.47]**	3.12 [0.99, 7.67]	0.93 [0.23, 2.52]
0.79 [0.28, 1.67]	0.44 [0.09, 1.22]	0.99 [0.26, 2.58]	**OLA**	**1.76 [1.06, 2.75]**	1.81 [0.81, 3.57]	0.55 [0.18, 1.40]
**0.61 [0.35, 0.99]**	**0.34 [0.10, 0.83]**	0.77 [0.29, 1.69]	0.95 [0.30, 2.46]	**QUE**	1.05 [0.50, 2.00]	**0.32 [0.11, 0.80]**
NA	NA	NA	NA	NA	**ZIP**	**0.33 [0.09, 0.87]**
2.36 [0.39, 8.83]	1.31 [0.14, 5.39]	2.96 [0.39, 11.82]	3.74 [0.43, 15.93]	4.17 [0.61, 16.11]	NA	**LUR**

Note: Somnolence results are on the top-right and Switch to Mania results are on the bottom-left. Numbers represent the odds ratio [95% credible interval]. In the top-right comparisons, the row treatment is the reference category

*Abbreviations*: *PLO* Placebo, *ARI* Aripiprazole, *CAR* Cariprazine, *LUR* Lurasidone, *OLA* Olanzapine, *QUE* Quetiapine, *ZIP* Ziprasidone

**Table 13:** Odds Ratios for Extrapyramidal Symptoms and Akathisia

**PLO**	1.98 [0.96, 3.64]	**2.26 [1.26, 3.83]**	**2.76 [1.61, 4.58]**	**4.12 [1.05, 12.76]**
**12.15 [2.07,39.95]**	**ARI**	1.28 [0.49, 2.77]	1.57 [0.62, 3.38]	2.33 [0.47,7.85]
**3.79 [1.19,9.27]**	0.57 [0.06,2.23]	**CAR**	1.33 [0.58, 2.66]	1.98 [0.43, 6.51]
**N/A**	N/A	N/A	**QUE**	1.60 [0.35, 5.27]
9.50 [0.67,36.33]	1.45 [0.04,6.36]	6.61 [0.15,13.84]	N/A	**LUR**

Note: Extrapyramidal Symptom results are on the top-right and Akathisia results are on the bottom-left. Results give the odds ratio [95% credible interval]. In the top-right comparisons, the row treatment is the reference treatment. In the bottom-left, the column treatment is the reference category

*Abbreviations*: *PLO* Placebo, *ARI* Aripiprazole, *CAR* Cariprazine, *LUR* Lurasidone, *QUE* Quetiapine

**Table 14:** Surface Under the Cumulative Ranking Curve (SUCRA)

	Lurasidone	Cariprazine	Olanzapine	Quetiapine	Aripiprazole	Ziprasidone	Placebo
Outcome	SUCRA (Rank)	SUCRA (Rank)	SUCRA (Rank)	SUCRA (Rank)	SUCRA (Rank)	SUCRA (Rank)	SUCRA (Rank)
**Efficacy Outcomes**							
MADRS	0.82 (2)	0.49 (4)	0.81 (2)	0.86 (2)	0.28 (6)	0.27 (5)	0.04 (7)
CGI-BP-S overall	0.85 (1)	0.45 (4)	0.63 (3)	0.75 (2)	N/A	0.19 (5)	0.10 (6)
CGI-BP-S depression	0.95 (1)	N/A	0.58 (2)	N/A	0.41 (3)	N/A	0.06 (4)
Response	0.96 (1)	0.54 (4)	0.6 (3)	0.86 (2)	0.23 (5)	0.2 (6)	0.1 (6)
Remission (MADRS ≤12)	0.87 (1)	N/A	0.50 (3)	0.84 (2)	N/A	0.14 (4)	0.11 (5)
Remission (MADRS ≤10)	0.88 (1)	0.60 (2)	N/A	N/A	N/A	N/A	0.00 (3)
**Discontinuation Outcomes**							
All-Cause	0.51 (4)	0.53 (4)	0.96 (1)	0.62 (3)	0.07 (7)	0.21 (6)	0.6 (3)
Due to Efficacy	0.45 (4)	0.61 (3)	0.70 (3)	0.94 (1)	0.50 (4)	0.07 (7)	0.19 (6)
Due to Adverse Events	0.77 (2)	0.52 (4)	0.56 (4)	0.13 (6)	0.18 (6)	0.5 (4)	0.84 (2)
**Metabolic Outcomes**							
Change in Body Weight	0.66 (3)	0.45 (4)	0.0 (6)	0.20 (5)	0.75 (2)	N/A	0.91 (1)
≥ 7% Weight Gain	0.37 (5)	0.47 (4)	0.01 (6)	0.42 (4)	0.77 (2)	N/A	0.94 (1)
Change in glucose	0.78 (1)	0.53 (3)	0.61 (2)	0.20 (5)	0.31 (5)	N/A	0.54 (3)
Change in Triglycerides	0.76 (1)	0.49 (4)	0.40 (4)	0.09 (6)	0.53 (3)	N/A	0.70 (2)
Change in Cholesterol	0.44 (4)	0.84 (1)	0.04 (6)	0.54 (3)	0.54 (3)	N/A	0.58 (3)
Change in LDL	0.30 (6)	0.70 (2)	0.29 (5)	0.60 (2)	0.59 (2)	N/A	0.49 (4)
**Other Outcomes**							
Prolactin	0.01 (5)	0.30 (4)	N/A	0.58 (3)	0.73 (2)	N/A	0.85 (1)
Somnolence	0.76 (2)	0.64 (3)	0.38 (5)	0.09 (7)	0.59 (3)	0.11 (6)	0.94 (1)
EPS	0.22 (5)	0.45 (3)	N/A	0.28 (4)	0.57 (2)	N/A	0.99 (1)
Akathisia	0.13 (4)	0.38 (3)	N/A	N/A	0.51 (2)	N/A	0.97 (1)
Switch to Mania	0.74 (5)	0.41 (3)	0.29 (2)	0.13 (1)	0.88 (6)	NA	0.55 (4)

### NNT/NNH

Descriptive estimates of the number needed to treat (NNT) to achieve one additional responder relative to placebo and the number needed to harm (NNH) based on one additional all-cause discontinuation and discontinuation due to adverse events relative to placebo were calculated for each treatment arm. Lurasidone (5) had the lowest NNT value for response (highest responder rates) followed by quetiapine (6), olanzapine (10), cariprazine (11), aripiprazole (50) and ziprasidone (100). For remission defined as MADRS ≤12 lurasidone (6) and quetiapine (6) had lowest NNT followed by olanzapine (13) and ziprasidone (250). For remission defined as MADRS ≤10 lurasidone (8) had lower NNT than cariprazine (13). The NNH values for all-cause discontinuations were the highest for quetiapine (500) (lowest discontinuation rate) followed by lurasidone (100), cariprazine (100), olanzapine (15), ziprasidone (15) and aripiprazole (10). The NNH for discontinuation due to adverse events was the highest for lurasidone (250) followed by cariprazine (50), olanzapine (50), ziprasidone (50), aripiprazole (17) and quetiapine (15).

### Sensitivity analyses

Dose stratified sensitivity analysis results were largely similar to the base case findings. For cariprazine, only the 1.5 mg and 3.0 mg cariprazine treatment arms used once daily were significantly greater than placebo, whereas all the stratified doses for lurasidone, olanzapine, and quetiapine were significantly more efficacious than placebo.

### Quality of evidence and heterogeneity

Overall, the quality of evidence for the primary outcome was high for direct evidence with quality reduced for NMA evidence, primarily because of indirectness of results and imprecisions. For all other outcomes, in general, lurasidone vs placebo and cariprazine vs placebo comparisons had higher quality, whereas ziprasidone vs placebo, and aripiprazole vs placebo comparisons had lower quality owing to limitations in the risk of bias. GRADE results are presented in Additional file 1: Table 7a-b.

Overall, the risk of bias was low, with 6 studies showing some concerns related to the randomization process (e.g., incomplete description of allocation concealment). Results of the risk of bias assessment is presented in Additional file 1: Figure 2.

For the primary outcome change from baseline in MADRS, comparison-adjusted funnel plots of the network meta-analysis did not suggest that small studies gave different results from larger studies. This was true for all other outcomes evaluated in this network meta-analysis, except for continuous outcomes change from baseline in CGI-BP-S overall, and triglycerides, and dichotomous outcomes of response, ≥7% weight gain, and EPS. Potential asymmetry was detected in the funnel plots for these 5 outcomes, suggesting a possibility of reporting bias. Funnel plots can be found in the Additional file 1: Figure 3a-d. Assessment of transitivity showed most of the studies and comparisons had minimal variation in mean age, sex, MADRS and CGI-BP-S depression score at baseline results. Other effect modifiers such as age at onset, weight, and BMI at baseline were reported in few studies only. Most of the studies included only BPD-I patients, whereas 7 (39%) studies included both BPD-I and BPD-II patients. Detailed results are presented in Additional file 1: Figure 4a, b. Heterogeneity was assessed by the median between-study variance (Tau^2^) and ranged from 0 to 12.57, with some considered moderate to high (Additional file 1: Table 8).

## Discussion

In this NMA involving short term trials of atypical antipsychotic monotherapy treatment for patients with bipolar depression, lurasidone, quetiapine, olanzapine, and cariprazine were found to be significantly more efficacious than placebo as assessed by change in MADRS. In pairwise comparisons for change in MADRS, lurasidone, cariprazine, olanzapine, and quetiapine were found to be similar. According to SUCRA analyses lurasidone, olanzapine and quetiapine ranked first for improvement in MADRS compared to placebo followed by cariprazine, ziprasidone and aripiprazole.

While mean change from baseline on MADRS is often a primary endpoint in clinical trials, clinicians in practice are often also interested in understanding the magnitude of treatment response. Lurasidone had significantly greater odds of response (defined as ≥50% improvement in MADRS), than cariprazine, aripiprazole, and ziprasidone. In addition, the NNT for response was the lowest for lurasidone when compared to other atypical antipsychotics. With lurasidone, 5 patients needed to be treated for one patient to respond, while other atypical antipsychotics required treating 6 (quetiapine) to 12 (cariprazine) patients for one patient to respond.

For improving overall severity assessed by CGI-BP-S, lurasidone, quetiapine and cariprazine were significantly more efficacious than placebo; and in pairwise comparisons for overall CGI-BP-S, lurasidone was associated with significantly more improvement than cariprazine and ziprasidone. Lurasidone was significantly better than placebo for improvement in CGI-BP-S depression score, but olanzapine and aripiprazole were not. CGI-S scores represent a global assessment of patient severity by the investigator and therefore provide a clinically relevant measure of real-world effectiveness.

Lurasidone and aripiprazole had similar weight gain compared to placebo while cariprazine, olanzapine and quetiapine had significantly greater weight gain than placebo. Additionally, lurasidone also had significantly less weight gain than olanzapine and quetiapine. Lurasidone had highest NNH values (lowest rates) for discontinuation due to adverse events. The current NMA extends earlier meta-analyses by including cariprazine, conducting pairwise comparisons between all atypical antipsychotics, examining additional outcome variables such as triglycerides, LDL, glucose, prolactin, and assessing discontinuation due to efficacy and adverse events.

The efficacy findings for lurasidone, quetiapine, and olanzapine are consistent with previous meta-analyses of atypical antipsychotic monotherapy in bipolar depression [12–14, 16, 48–52]. While prior meta-analyses have largely focused on efficacy instead of tolerability, estimated differences in all-cause discontinuation in prior analyses have also been consistent with the current analyses [14, 15].

Antidepressant monotherapy appears to be the most common treatment in bipolar depression in usual clinical care, despite treatment guidelines highlighting the lack of evidence supporting their use and recognizing concern about switching patients into mania [49–53]. Given the limited evidence for efficacy and the potential to switch to mania or cause rapid cycling [11, 54] this practice appears to be a potential target for further evidence based investigation. Consistent with accumulating evidence of the efficacy of some atypical antipsychotics in bipolar depression, the use of atypical antipsychotics has been increasing in bipolar disorder [55, 56].

### Limitations

There were several limitations to this NMA. The quetiapine trials included bipolar II patients which may confound the results. While the baseline patient characteristics that were examined from the different trials appeared largely similar, unmeasured confounders could exist. Another limitation was inconsistent reporting of outcome variables. Some outcome variables such as EPS symptoms were not reported for all trials or not reported consistently [35, 36, 39, 44–46]. In addition, the metabolic laboratory values from the different studies were not all specified as fasting measurements. To increase consistency in study design and reported outcomes, this NMA was limited to atypical antipsychotics used as monotherapy to treat patients with bipolar depression. Inclusion of other treatments such as lithium, lamotrigine, and divalproex as well as combination treatments was beyond the scope of the current analysis. Although the included studies were deemed comparable in transitivity assessment, moderate to high heterogeneity was observed for some outcomes in the NMA, compared with the predictive distribution of heterogeneity [31]. Similar to the original NMA, meta-regression, which can potentially adjust for effect modifiers, was not performed because of the absence of a sufficiently large number of trials per comparison that is required to render meta-regression feasible at the aggregate level [15]. Random effects models were applied to account for between-study variance for the NMA, but the presence of heterogeneity should be acknowledged when interpreting the findings. For the included evidence, despite the statistical tests showing no small-study effects for most outcomes, we found some potential asymmetry of comparison-adjusted funnel plots in this network meta-analysis. All the identified trials were placebo controlled RCTs leading to star shaped networks, and therefore indirect evidence and hierarches should be interpreted with caution.

## Conclusions

In this NMA in adults with bipolar depression, which evaluated change in depressive symptoms (assessed by MADRS) across short-term trials, the largest improvement versus placebo was observed for lurasidone, olanzapine and quetiapine with cariprazine, showing smaller treatment effect. Aripiprazole and ziprasidone were ineffective for the treatment of bipolar depression.. Improvement in CGI-BP-S score for lurasidone was larger than cariprazine and ziprasidone but similar to quetiapine and olanzapine. Based on short term studies lurasidone and aripiprazole had similar weight gain compared to placebo.

## Supplementary Information

**Additional file 1: Table 1.** The PRISMA 2020 Statement. **Table 2a.** Search Strings. **Table 2b.** Conference Proceedings Reviewed. **Table 3a.** Fixed effect model for Change from Baseline in MADRS and Odds Ratio for Response. **Table 3b.** Fixed effect model for Change from Baseline in CGI-BP-S-depression and CGI-BP-S-overall. **Table 3c.** Fixed effect model for Odds Ratios for Remission (MADRS ≤ 12) and Remission (MADRS ≤ 10). **Table 4a.** Fixed effect model for Odds Ratios for All-Cause Discontinuation and Discontinuation Due to Adverse Events. **Table 4b.** Fixed effect model for Odds Ratios for Discontinuation Due Lack of Efficacy. **Table 5a.** Fixed effect model for Change from Baseline in Weight and Odds Ratios of ≥ 7% Weight Gain. **Table 5b.** Fixed effect model for Change from Baseline in Triglycerides and Total Cholesterol. **Table 5c.** Fixed effect model for Change from Baseline in Low-Density Lipoprotein Cholesterol and Glucose. **Table 5d.** Fixed effect model for Change from Baseline in Prolactin. **Table 6a.** Fixed effect model for Odds Ratios for Somnolence and Switch to Mania. **Table 6b.** Fixed effect model for Odds Ratios for Extrapyramidal Symptoms and Akathisia. **Table 7a.** GRADE assessment of Continuous Outcomes. **Table 7b.** GRADE assessment of Dichotomous Outcomes. **Table 8.** Heterogeneity Assessment through Tau^2^ of the networks. **Figure 1a.** Network Diagrams for Change from Baseline in MADRS, CGI-BP-S Overall, CGI-BP-S-Depression, Weight and Blood Glucose. **Figure 1b.** Network Diagrams for Change from Baseline in Triglycerides, Total Cholesterol, Low-Density Lipoprotein Cholesterol and Prolactin. **Figure 1c.** Network Diagrams for Response, Remission (MADRS ≤ 12 and ≤ 10), All Cause Discontinuation, Discontinuation due to Lack of Efficacy and Discontinuation due to Adverse Events. **Figure 1d.** Network Diagrams for ≥ 7% weight gain, Akathisia, Switch to Mania, Extrapyramidal Symptoms and Somnolence. **Figure 2.** Risk of Bias Assessment of Included Studies. **Figure 3a.** Assessment of Publication Bias Through Funnel Plots for Change from Baseline in MADRS, CGI-BP-S Overall, CGI-BP-S-Depression, Weight and Blood Glucose. **Figure 3b.** Assessment of Publication Bias Through Funnel Plots for Triglycerides, Total Cholesterol, Low-Density Lipoprotein Cholesterol and Prolactin. **Figure 3c.** Assessment of Publication Bias Through Funnel Plots for Response, Remission (MADRS ≤ 12 and ≤ 10), All Cause Discontinuation, Discontinuation due to Lack of Efficacy and Discontinuation due to Adverse Events. **Figure 3d.** Assessment of Publication Bias Through Funnel Plots for ≥ 7% weight gain, Akathisia, Switch to Mania, Extrapyramidal Symptoms and Somnolence. **Figure 4a.** Heterogeneity Assessment of Included RCTs Through Effect Modifier Comparison. **Figure 4b.** Heterogeneity Assessment of Included RCTs Through Effect Modifier Comparison (Contd.).

## Data Availability

The datasets used and/or analyzed during the current study available from the corresponding author on reasonable request.

## Abbreviations

CGI-BP-S: Clinical Global Impression Scale Bipolar Version – Severity
CANMAT: Canadian Network for Mood and Anxiety Treatment
EPS: Extrapyramidal Symptoms
FDA: US Food and Drug Administration
GRADE: The Grading of Recommendation, Assessment, Development and Evaluation
ISBD: International Society for Bipolar Disorders guidelines
LDL: Low-Density Lipoprotein
MADRS: Montgomery Åsberg Depression Rating Scale
MMRM: Mixed Models for Repeated Measures
NMA/NMAs: Network Meta-Analysis/Network Meta-Analyses
NNH: Number Needed to Harm
NNT: Number Needed to Treat
OR: Odds Ratios
PICOS: Patient, Intervention, Comparator, Outcome, and Study
PRISMA: Preferred Reporting Items for Systematic Reviews and Meta-Analyses
RCT: Randomized Controlled Trials
SUCRA: Surface under the cumulative ranking curve
US: United States
XR: Extended Release
