# Supplementary material for: Efficacy and tolerability of atypical antipsychotics for acute bipolar depression: a network meta-analysis
Source: BMC Psychiatry. 2021 May 11;21:249. doi: 10.1186/s12888-021-03220-3 (PMC8112003; doi:10.1186/s12888-021-03220-3)
Supplement: Supplementary file 1 — Additional file 1: Table 1. The PRISMA 2020 Statement. Table 2a. Search Strings. Table 2b. Conference Proceedings Reviewed. Table 3a. Fixed effect model for Change from Baseline in MADRS and Odds Ratio for Response. Table 3b. Fixed effect model for Change from Baseline in CGI-BP-S-depression and CGI-BP-S-overall. Table 3c. Fixed effect model for Odds Ratios for Remission (MADRS ≤ 12) and Remission (MADRS ≤ 10). Table 4a. Fixed effect model for Odds Ratios for All-Cause Discontinuation and Discontinuation Due to Adverse Events. Table 4b. Fixed effect model for Odds Ratios for Discontinuation Due Lack of Efficacy. Table 5a. Fixed effect model for Change from Baseline in Weight and Odds Ratios of ≥ 7% Weight Gain. Table 5b. Fixed effect model for Change from Baseline in Triglycerides and Total Cholesterol. Table 5c. Fixed effect model for Change from Baseline in Low-Density Lipoprotein Cholesterol and Glucose. Table 5d. Fixed effect model for Change from Baseline in Prolactin. Table 6a. Fixed effect model for Odds Ratios for Somnolence and Switch to Mania. Table 6b. Fixed effect model for Odds Ratios for Extrapyramidal Symptoms and Akathisia. Table 7a. GRADE assessment of Continuous Outcomes. Table 7b. GRADE assessment of Dichotomous Outcomes. Table 8. Heterogeneity Assessment through Tau2 of the networks. Figure 1a. Network Diagrams for Change from Baseline in MADRS, CGI-BP-S Overall, CGI-BP-S-Depression, Weight and Blood Glucose. Figure 1b. Network Diagrams for Change from Baseline in Triglycerides, Total Cholesterol, Low-Density Lipoprotein Cholesterol and Prolactin. Figure 1c. Network Diagrams for Response, Remission (MADRS ≤ 12 and ≤ 10), All Cause Discontinuation, Discontinuation due to Lack of Efficacy and Discontinuation due to Adverse Events. Figure 1d. Network Diagrams for ≥ 7% weight gain, Akathisia, Switch to Mania, Extrapyramidal Symptoms and Somnolence. Figure 2. Risk of Bias Assessment of Included Studies. Figure 3a. Assessment of Publication Bias Throug [file 12888_2021_3220_MOESM1_ESM.docx]

Efficacy and Tolerability of Atypical Antipsychotics for Acute Bipolar Depression: A Network Meta-Analysis

Aditi Kadakia^1^, Carole Dembek^1^, Vincent Heller^2^, Rajpal Singh^3^, Jennifer Uyei,^4^ Katsuhiko Hagi^5^, Tadashi Nosaka^5^, and Antony Loebel^1^

1 Sunovion Pharmaceuticals Inc., 84 Waterford Dr, Marlborough, MA 01752

2 IQVIA, Stockholm, Sweden

3 IQVIA, Mumbai, India

4 IQVIA, San Francisco, USA

5 Sumitomo Dainippon Pharma Co., Ltd., Tokyo, Japan

Corresponding Author: Aditi Kadakia, Aditi.Kadakia@sunovion.com

Journal: BMC Psychiatry

**Appendix Table 1. The PRISMA 2020 Statement**

Checklist of items to include when reporting a systematic review involving a network meta-analysis

| **Section and Topic** | **Item #** | **Checklist item** | **Location where item is reported** |
| --- | --- | --- | --- |
| **TITLE** | | |  |
| Title | 1 | Identify the report as a systematic review. | Page 1 |
| **ABSTRACT** | | |  |
| Abstract | 2 | See the PRISMA 2020 for Abstracts checklist. | Page 3-4 |
| **INTRODUCTION** | | |  |
| Rationale | 3 | Describe the rationale for the review in the context of existing knowledge. | Page 6 |
| Objectives | 4 | Provide an explicit statement of the objective(s) or question(s) the review addresses. | Page 7 |
| **METHODS** | | |  |
| Eligibility criteria | 5 | Specify the inclusion and exclusion criteria for the review and how studies were grouped for the syntheses. | Table 1 |
| Information sources | 6 | Specify all databases, registers, websites, organisations, reference lists and other sources searched or consulted to identify studies. Specify the date when each source was last searched or consulted. | Page 7-8 |
| Search strategy | 7 | Present the full search strategies for all databases, registers and websites, including any filters and limits used. | Appendix Table 2a |
| Selection process | 8 | Specify the methods used to decide whether a study met the inclusion criteria of the review, including how many reviewers screened each record and each report retrieved, whether they worked independently, and if applicable, details of automation tools used in the process. | Page 8 |
| Data collection process | 9 | Specify the methods used to collect data from reports, including how many reviewers collected data from each report, whether they worked independently, any processes for obtaining or confirming data from study investigators, and if applicable, details of automation tools used in the process. | Page 8 |
| Data items | 10a | List and define all outcomes for which data were sought. Specify whether all results that were compatible with each outcome domain in each study were sought (e.g. for all measures, time points, analyses), and if not, the methods used to decide which results to collect. | Page 9 |
|  | 10b | List and define all other variables for which data were sought (e.g. participant and intervention characteristics, funding sources). Describe any assumptions made about any missing or unclear information. | Page 9 |
| Study risk of bias assessment | 11 | Specify the methods used to assess risk of bias in the included studies, including details of the tool(s) used, how many reviewers assessed each study and whether they worked independently, and if applicable, details of automation tools used in the process. | Page 8 |
| Effect measures | 12 | Specify for each outcome the effect measure(s) (e.g. risk ratio, mean difference) used in the synthesis or presentation of results. | Page 9-11 |
| Synthesis methods | 13a | Describe the processes used to decide which studies were eligible for each synthesis (e.g. tabulating the study intervention characteristics and comparing against the planned groups for each synthesis (item #5)). | Page 12-13 |
|  | 13b | Describe any methods required to prepare the data for presentation or synthesis, such as handling of missing summary statistics, or data conversions. | Page 10 |
|  | 13c | Describe any methods used to tabulate or visually display results of individual studies and syntheses. | Page 10-12 |
|  | 13d | Describe any methods used to synthesize results and provide a rationale for the choice(s). If meta-analysis was performed, describe the model(s), method(s) to identify the presence and extent of statistical heterogeneity, and software package(s) used. | Page 10-12 |
|  | 13e | Describe any methods used to explore possible causes of heterogeneity among study results (e.g. subgroup analysis, meta-regression). | Page 12-13 |
|  | 13f | Describe any sensitivity analyses conducted to assess robustness of the synthesized results. | Page 12 |
| Reporting bias assessment | 14 | Describe any methods used to assess risk of bias due to missing results in a synthesis (arising from reporting biases). | Page 12-13 |
| Certainty assessment | 15 | Describe any methods used to assess certainty (or confidence) in the body of evidence for an outcome. | Page 12-13 |
| **RESULTS** | | |  |
| Study selection | 16a | Describe the results of the search and selection process, from the number of records identified in the search to the number of studies included in the review, ideally using a flow diagram. | Page 13-14; Figure 1 |
|  | 16b | Cite studies that might appear to meet the inclusion criteria, but which were excluded, and explain why they were excluded. | Page 13-14 |
| Study characteristics | 17 | Cite each included study and present its characteristics. | Page 14; Table 2 |
| Risk of bias in studies | 18 | Present assessments of risk of bias for each included study. | Page 20; Appendix Figure 2 |
| Results of individual studies | 19 | For all outcomes, present, for each study: (a) summary statistics for each group (where appropriate) and (b) an effect estimate and its precision (e.g. confidence/credible interval), ideally using structured tables or plots. | Table 3a-6b |
| Results of syntheses | 20a | For each synthesis, briefly summarise the characteristics and risk of bias among contributing studies. | Page 20 |
|  | 20b | Present results of all statistical syntheses conducted. If meta-analysis was done, present for each the summary estimate and its precision (e.g. confidence/credible interval) and measures of statistical heterogeneity. If comparing groups, describe the direction of the effect. | Page 15-20 |
|  | 20c | Present results of all investigations of possible causes of heterogeneity among study results. | Page 20-21 |
|  | 20d | Present results of all sensitivity analyses conducted to assess the robustness of the synthesized results. | Page 20 |
| Reporting biases | 21 | Present assessments of risk of bias due to missing results (arising from reporting biases) for each synthesis assessed. | Page 20-21; Appendix Table 3a-3b |
| Certainty of evidence | 22 | Present assessments of certainty (or confidence) in the body of evidence for each outcome assessed. | Page 20-21; Appendix Table 7a-7b |
| **DISCUSSION** | | |  |
| Discussion | 23a | Provide a general interpretation of the results in the context of other evidence. | Page 21-22 |
|  | 23b | Discuss any limitations of the evidence included in the review. | Page 23 |
|  | 23c | Discuss any limitations of the review processes used. | Page 23 |
|  | 23d | Discuss implications of the results for practice, policy, and future research. | Page 23 |
| **OTHER INFORMATION** | | |  |
| Registration and protocol | 24a | Provide registration information for the review, including register name and registration number, or state that the review was not registered. | Review was not registered |
|  | 24b | Indicate where the review protocol can be accessed, or state that a protocol was not prepared. | Protocol attached in Appendix section |
|  | 24c | Describe and explain any amendments to information provided at registration or in the protocol. | Protocol amendments listed in Appendix section |
| Support | 25 | Describe sources of financial or non-financial support for the review, and the role of the funders or sponsors in the review. | Page 3 |
| Competing interests | 26 | Declare any competing interests of review authors. | Page 2 |
| Availability of data, code and other materials | 27 | Report which of the following are publicly available and where they can be found: template data collection forms; data extracted from included studies; data used for all analyses; analytic code; any other materials used in the review. | Aditi.Kadakia@sunovion.com |

**Appendix Table 2a.** **Search Strings**

| **#** | **Searches** | **Results** |
| --- | --- | --- |
|  | **Disease Terms** |  |
| 1 | exp bipolar disorder/ or exp mania/ | 145955 |
| 2 | ((bi?polar adj5 (disorder$ or depress$)) or ((cyclothymi$ or rapid or ultradian) adj5 cycl$) or hypomani$ or mania$ or manic$ or mixed episode$ or rcbd).mp. | 217591 |
| 3 | Or/1-2 | 217591 |
|  | **Study Design Terms** |  |
| 4 | exp "clinical trial (topic)"/ or exp clinical trial/ | 2656190 |
| 5 | ("crossover procedure" or "double blind procedure" or placebo or randomization or "random sample" or "single blind procedure").mp. | 1444532 |
| 6 | (((single$ or doubl$ or trebl$ or tripl$) adj2 blind$) or mask$ or dummy or doubleblind$ or singleblind$ or trebleblind$ or tripleblind$).mp. | 1166714 |
| 7 | (placebo$ or random$ or treatment outcome$).mp. | 6336264 |
| 8 | exp animals/ not human$.mp. | 8922443 |
| 9 | (or/4-7) not 8 | 7200974 |
|  | **Intervention Related Terms** |  |
| 10 | exp lurasidone/ or (latuda or "SM 13,496" or "SM-13496" or "SM 13496").mp. | 1865 |
| 11 | exp cariprazine/ or (RGH-188 or "RGH 188" or Vraylar or Reagila).mp. | 575 |
| 12 | exp quetiapine/ or ("quetiapine fumarate" or seroquel or temprolide or socalm or tienapine or "ICI 204,636" or "ICI 204636" or "ICI-204636").mp. | 29524 |
| 13 | exp olanzapine/ or (Zyprexa or Zypadhera or Olansek or Zalasta or "Lanzek Zydis Wafers" or anzartric or dopin or joylon or lanopin or lanzac or meltolan or midax or olace or oladay or olan or olandus or olanex or olapin or olazax or oleanz or olexar or oltal or olzap or onza or ozapin or psychozap or relprevv or zelta or zydis or zyprex or zyprexav or "LY-170053" or "LY 170053").mp. | 47154 |
| 14 | exp Aripiprazole/ or (Aripiprazole or Abilify or OPC-14597 or "OPC 14597").mp. | 24267 |
| 15 | exp Asenapine/ or (Saphris or Sycrest or "ORG 5222" or "ORG 5222").mp. | 1573 |
| 16 | exp Risperidone/ or (Risperdal or belivon or consta or neripros or noprenia or riperidon or risolept or rispen or rispid or rispolet or rizodal or sequinan or zargus or zofredal or "R 64766" or "R-64,766" or R64766).mp. | 47959 |
| 17 | exp Ziprasidone/ or (Geodon or Zeldox or Zipwell or zeldrox or zipsydon or "CP 88059" or "CP-88,059" or " CP-88059").mp. | 9070 |
| 18 | exp brexpiprazole/ or (Rexulti or "OPC-34712" or "OPC 34712" or OPC34712).mp. | 552 |
| 19 | exp Lumateperone/ or (ITI-722 or iti722 or "iti 722" or "iti-007" or "iti 007" or "iti007").mp. | 138 |
| 20 | exp atypical antipsychotic/ or exp antipsychotic/ or antipsychotic.mp. | 32369 |
| 21 | or/10-20 | 241430 |
|  | **Disease, Study Design, and Intervention Terms with Restrictions** |  |
| 22 | 3 and 9 and 21 | 10981 |
| 23 | limit 22 to English | 10164 |
| 24 | limit 23 to yr="2015-current" | 2471 |
| 25 | remove duplicates from 24 | 1791 |

**Appendix Table 2b:** **Conference Proceedings Reviewed**

In addition to the medical literature databases, a few websites were searched for conference abstracts (previous year only) reporting early results from recently completed randomized controlled trials from the following sources:

- International Society for Bipolar Disorders (<https://onlinelibrary.wiley.com/toc/13995618/2020/22/2>)
- International College of Neuropsychopharmacology (<https://cinp.org/Past-Events>)
- American Psychiatric Association (<https://www.psychiatry.org/psychiatrists/meetings/annual-meeting/guide>)
- U.S. Psychiatric & Mental Health (<https://www.psychcongress.com/posters?field_pcn_year_tid=681&title>)
- American Society of Clinical Psychopharmacology (<https://pmg.joynadmin.org/documents/1005/5ce6b5d068ed3f515b95e300.pdf>)
- American College of Neuropsychopharmacology (<https://www.nature.com/collections/dcgjichbhj> )
- American Academy of Child and Adolescent Psychiatry (<https://www.jaacap.org/issue/S0890-8567(20)X0002-5>)
- European College of Neuropsychopharmacology (<https://www.ecnp.eu/about-ecnp/history/past-ecnp-meetings/past-congresses/Copenhagen2019>)
- European Congress of Psychiatry (http://archive.epa congress.org/2019/Pages/default.html )
- European Psychiatric Association (<http://archive.epa-congress.org/2019/abstracts/abstract-book-2018.html>)
- Neuroscience Education Institute (<https://www.cambridge.org/core/journals/cns-spectrums/issue/121F11AF363714B4E6027BAAA628E6D4>)

**Appendix Table 3a. Fixed effect model for Change from Baseline in MADRS and Odds Ratio for Response**

| **PLO** | -1.07 [-3.04 ,0.89] | **-2.33 [-3.36,-1.29]** | **-4.64 [-5.79,-3.49]** | **-4.82 [-5.79,-3.85]** | -1.32 [-3.11, 0.47] | **-4.70 [-6.69,-2.69]** |
| --- | --- | --- | --- | --- | --- | --- |
| 1.10 [0.80, 1.47] | **ARI** | -1.26 [-3.48, 0.97] | **-3.57 [-5.84,-1.30]** | **-3.75 [-5.94,-1.55]** | -0.25 [-2.90, 2.40] | **-3.63 [-6.43,-0.81]** |
| **1.47 [1.18, 1.80]** | 1.36 [0.93, 1.94] | **CAR** | **-2.31 [-3.87,-0.76]** | **-2.49 [-3.92,-1.07]** | 1.01 [-1.06, 3.07] | **-2.37 [-4.62,-0.12]** |
| **1.54 [1.22, 1.94]** | 1.44 [0.96, 2.07] | 1.07 [0.77, 1.44] | **OLA** | -0.18 [-1.69, 1.33] | **3.32 [1.19, 5.45]** | -0.06 [-2.38, 2.25] |
| **2.08 [1.75, 2.46]** | **1.94 [1.35, 2.69]** | **1.44 [1.09, 1.86]** | **1.37 [1.02, 1.81]** | **QUE** | **3.50 [1.47, 5.53]** | 0.12 [-2.11, 2.35] |
| 1.08 [0.79, 1.45] | 1.01 [0.64, 1.51] | 0.75 [0.51, 1.06] | 0.71 [0.48, 1.02] | **0.52 [0.36, 0.73]** | **ZIP** | **-3.38 [-6.07,-0.71]** |
| **2.57 [1.69, 3.79]** | **2.39 [1.41, 3.84]** | **1.77 [1.10, 2.72]** | **1.69 [1.04, 2.62]** | 1.24 [0.79, 1.89] | **2.43 [1.42, 3.91]** | **LUR** |

Note: MADRS results are on the top-right and response results are on the bottom-left. Results give the mean change and odds ratio [95% credible interval]. In the top-right comparisons, the row treatment is the reference category. In the bottom-left, the column treatment is the reference category. Response was defined as ≥ 50% improvement in MADRS.

Abbreviations: MADRS - Montgomery–Åsberg Depression Rating Scale; PLO – Placebo; ARI - Aripiprazole; CAR – Cariprazine; LUR – Lurasidone; OLA – Olanzapine; QUE – Quetiapine; ZIP – Ziprasidone

**Appendix Table 3b. Fixed effect model for Change from Baseline in CGI-BP-S-depression and CGI-BP-S-overall**

| **PLO** | NA | **-0.25 [-0.37, -0.13]** | -0.62 [-2.11, 0.87] | **-0.55 [-0.68, -0.43]** | -0.04 [-0.25, 0.17] | **-0.63 [-0.84, -0.41]** |
| --- | --- | --- | --- | --- | --- | --- |
| -0.21 [-0.43, 0.01] | **ARI** | NA | NA | NA | NA | NA |
| NA | NA | **CAR** | -0.37 [-1.87, 1.13] | **-0.30 [-0.48, -0.13]** | 0.21 [-0.04, 0.45] | **-0.37 [-0.62, -0.13]** |
| **-0.32 [-0.51, -0.12]** | -0.11 [-0.40, 0.18] | NA | **OLA** | 0.07 [-1.43, 1.57] | 0.58 [-0.93, 2.08] | 0.00 [-1.51, 1.50] |
| NA | NA | NA | NA | **QUE** | **0.51 [0.26, 0.75]** | -0.07 [-0.32, 0.18] |
| NA | NA | NA | NA | NA | **ZIP** | **-0.58 [-0.88, -0.28]** |
| **-0.63 [-0.87, -0.39]** | **-0.42 [-0.74, -0.09]** | NA | -0.31 [-0.62, 0.00] | NA | NA | **LUR** |

Note: CGI-BP-S-overall are on the top-right and CGI-BP-S-depression results are on the bottom-left. Numbers represent the mean change [95% credible interval]. In the top-right comparisons, the row treatment is the reference category. In the bottom-left, the column treatment is the reference category.

Abbreviations: CGI-BP-S - Clinical Global Impressions–Bipolar–Severity Scale; PLO – Placebo; ARI - Aripiprazole; CAR – Cariprazine; LUR – Lurasidone; OLA – Olanzapine; QUE – Quetiapine; ZIP – Ziprasidone

**Appendix Table 3c. Fixed effect model for Odds Ratios for Remission (MADRS≤ 12) and Remission (MADRS≤ 10)**

| **PLO** | **1.60 [1.22, 2.06]** | NA | NA | NA | **2.12 [1.33, 3.27]** |
| --- | --- | --- | --- | --- | --- |
| NA | **CAR** | NA | NA | NA | 1.35 [0.78, 2.21] |
| **1.43 [1.12, 1.80]** | NA | **OLA** | NA | NA | NA |
| **2.04 [1.71, 2.41]** | NA | **1.45 [1.07, 1.92]** | **QUE** | NA | NA |
| 1.02 [0.76, 1.34] | NA | 0.73 [0.49, 1.03] | **0.51 [0.36, 0.69]** | **ZIP** | NA |
| **2.17 [1.40, 3.26]** | NA | 1.55 [0.93, 2.44] | 1.08 [0.67, 1.66] | **2.17 [1.27, 3.51]** | **LUR** |

Note: Remission defined as MADRS ≤ 10 results are on the top-right and Remission defined as MADRS ≤ 12 results are on the bottom-left. Numbers represent the odds ratio [95% credible interval]. In the top-right comparisons, the row treatment is the reference category. In the bottom-left, the column treatment is the reference category. Studies that used alternative definitions were not included: the aripiprazole studies and one quetiapine study defined remission as MADRS ≤8 at endpoint.

Abbreviations: PLO – Placebo; CAR – Cariprazine; LUR – Lurasidone; OLA – Olanzapine; QUE – Quetiapine; ZIP – Ziprasidone

**Appendix Table 4a. Fixed effect model for Odds Ratios for All-Cause Discontinuation and Discontinuation Due to Adverse Events**

| **PLO** | **1.66 [1.22, 2.21]** | 1.05 [0.81, 1.34] | **0.69 [0.54, 0.86]** | 0.99 [0.83, 1.18] | **1.37 [1.02, 1.80]** | 1.08 [0.69, 1.63] |
| --- | --- | --- | --- | --- | --- | --- |
| **2.39 [1.39, 3.90]** | **ARI** | **0.65 [0.43, 0.94]** | **0.42 [0.29, 0.61]** | **0.61 [0.43, 0.85]** | 0.84 [0.55, 1.24] | 0.67 [0.38, 1.09] |
| 1.41 [0.87, 2.19] | 0.63 [0.30, 1.18] | **CAR** | **0.67 [0.47, 0.92]** | 0.96 [0.70, 1.28] | 1.32 [0.89, 1.89] | 1.05 [0.62, 1.66] |
| 1.51 [0.96, 2.28] | 0.68 [0.32, 1.25] | 1.13 [0.57, 2.02] | **OLA** | **1.46 [1.08, 1.94]** | **2.02 [1.37, 2.86]** | 1.60 [0.95, 2.52] |
| **2.41 [1.73, 3.32]** | 1.08 [0.56, 1.89] | 1.81 [0.98, 3.05] | 1.68 [0.94, 2.78] | **QUE** | 1.39 [0.98, 1.90] | 1.10 [0.68, 1.70] |
| 1.49 [0.96, 2.25] | 0.67 [0.32, 1.23] | 1.12 [0.56, 1.99] | 1.04 [0.54, 1.82] | 0.64 [0.36, 1.05] | **ZIP** | 0.81 [0.47, 1.30] |
| 1.07 [0.47, 2.17] | 0.48 [0.17, 1.09] | 0.80 [0.30, 1.78] | 0.74 [0.28, 1.64] | **0.45 [0.18, 0.97]** | 0.75 [0.29, 1.66] | LUR |

Note: All-Cause Discontinuation results are on the top-right and Discontinuation Due to Adverse Events are in the bottom left. Results give the odds ratio [95% credible interval]. In the top-right comparisons, the row treatment is the reference category. In the bottom-left, the column treatment is the reference category.

Abbreviations: PLO – Placebo; ARI – Aripiprazole; CAR – Cariprazine; LUR – Lurasidone; OLA – Olanzapine; QUE – Quetiapine; ZIP – Ziprasidone

**Appendix Table 4b. Fixed effect model for Odds Ratios for Discontinuation Due Lack of Efficacy**

| **PLO** | 0.60 [0.30,1.08] | **0.51 [0.25,0.92]** | **0.48 [0.34,0.64]** | **0.23 [0.15,0.34]** | 1.60 [0.71,3.26] | 0.70 [0.31,1.40] |
| --- | --- | --- | --- | --- | --- | --- |
|  | **ARI** | 0.94 [0.34,2.11] | 0.88 [0.40,1.71] | **0.43 [0.18,0.86]** | 2.96 [0.97,7.19] | 1.30 [0.43,3.11] |
|  |  | **CAR** | 1.05 [0.47,2.01] | 0.51 [0.22,1.01] | **3.53 [1.15,8.55]** | 1.55 [0.50,3.69] |
|  |  |  | **OLA** | **0.50 [0.28,0.81]** | **3.46 [1.41,7.40]** | 1.52 [0.61,3.18] |
|  |  |  |  | **QUE** | **7.26 [2.79,16.04]** | **3.18 [1.23,6.92]** |
|  |  |  |  |  | **ZIP** | 0.51 [0.15,1.27] |
|  |  |  |  |  |  | **LUR** |

Note: All-Cause Discontinuation results are on the top-right. Numbers represent the odds ratio [95% credible interval]. In the top-right comparisons, the row treatment is the reference treatment. In the bottom-left, the column treatment is the reference category.

Abbreviations: PLO – Placebo; ARI - Aripiprazole; CAR – Cariprazine; LUR – Lurasidone; OLA – Olanzapine; QUE – Quetiapine; ZIP – Ziprasidone

**Appendix Table 5a. Fixed effect model for Change from Baseline in Weight and Odds Ratios of ≥7% Weight Gain**

| **PLO** | 0.19 [-0.54, 0.91] | **0.64 [0.41, 0.87]** | **2.91 [2.57, 3.24]** | **1.16 [0.90, 1.43]** | 0.35 [-0.03, 0.72] |
| --- | --- | --- | --- | --- | --- |
| 1.62 [0.70,3.27] | **ARI** | 0.45 [-0.30, 1.21] | **2.72 [1.92, 3.52]** | **0.98 [0.21, 1.74]** | 0.16 [-0.65, 0.97] |
| **3.26 [1.31,7.43]** | 2.34 [0.63,6.45] | **CAR** | **2.27 [1.86, 2.67]** | **0.53 [0.18, 0.87]** | -0.29 [-0.73, 0.14] |
| **64.35 [17.55,210.40]** | **46.27 [8.90,167.50]** | **24.09 [4.13,88.51]** | **OLA** | **-1.74 [-2.17, -1.31]** | **-2.56 [-3.06, -2.06]** |
| **3.43 [2.07,5.53]** | 2.46 [0.88,5.50] | 1.28 [0.40,2.94] | **0.08 [0.01,0.21]** | **QUE** | **-0.82 [-1.27, -0.36]** |
| 35.29 [0.75,142.00] | 25.20 [0.42,102.00] | 13.63 [0.20,52.81] | 0.85 [0.01,3.26] | 11.34 [0.21,43.59] | **LUR** |

Note: Weight Change results are on the top-right and numbers represent the mean change [95% credible interval]. ≥7% Weight Gain results are on the bottom-left and results give the odds ratio [95% credible interval]. In the top-right comparisons, the row treatment is the reference category. In the bottom-left, the column treatment is the reference category.

Abbreviations: PLO – Placebo; ARI – Aripiprazole; CAR – Cariprazine; LUR – Lurasidone; OLA – Olanzapine; QUE – Quetiapine

**Appendix Table 5b. Fixed effect model for Change from Baseline in Triglycerides and Total Cholesterol**

| **PLO** | 1.01 [-10.16, 12.24] | 0.18 [-0.06, 0.42] | **0.72 [0.21, 1.22]** | 10.84 [-3.11, 24.77] | -2.99 [-14.81, 8.73] |
| --- | --- | --- | --- | --- | --- |
| 0.30 [-3.19, 3.77] | **ARI** | -0.84 [-12.08, 10.34] | -0.29 [-11.53, 10.91] | 9.83 [-8.07, 27.81] | -4.01 [-20.29, 12.22] |
| 0.02 [-0.18, 0.22] | -0.28 [-3.76, 3.20] | **CAR** | 0.54 [-0.02, 1.10] | 10.66 [-3.29 ,24.59] | -3.17 [-14.99, 8.56] |
| **1.29 [0.63, 1.94**] | 0.99 [-2.55, 4.54] | **1.27 [0.58, 1.95]** | **OLA** | 10.12 [-3.84, 24.07] | -3.71 [-15.53, 8.01] |
| 0.43 [-3.45, 4.31] | 0.13 [-5.10, 5.32] | 0.40 [-3.48, 4.29] | -0.86 [-4.81, 3.08] | **QUE** | -13.83 [-32.08, 4.36] |
| 1.95 [-3.48, 7.40] | 1.65 [-4.80, 8.13] | 1.93 [-3.51, 7.38] | 0.66 [-4.82, 6.12] | 1.52 [-5.14, 8.18] | **LUR** |

Note: Triglycerides results are on the top-right and Total Cholesterol results are on the bottom-left. Numbers represent the mean change [95% credible interval]. In the top-right comparisons, the row treatment is the reference category. In the bottom-left, the column treatment is the reference category.

Abbreviations: PLO – Placebo; ARI – Aripiprazole; CAR – Cariprazine; LUR – Lurasidone; OLA – Olanzapine; QUE – Quetiapine

**Appendix Table 5c. Fixed effect model for Change from Baseline in Low-Density Lipoprotein Cholesterol and Glucose**

| **PLO** | -0.51 [-3.74, 2.73] | -0.08 [-0.23, 0.08] | **0.27 [0.15, 0.39]** | -0.59 [-3.93, 2.76] | 1.11 [-3.62, 5.80] |
| --- | --- | --- | --- | --- | --- |
| 0.78 [-1.88, 3.42] | **ARI** | 0.43 [-2.82, 3.66] | 0.77 [-2.47, 4.01] | -0.08 [-4.74, 4.58] | 1.62 [-4.09, 7.33] |
| -0.08 [-0.28, 0.13] | -0.86 [-3.51, 1.81] | **CAR** | **0.34 [0.15, 0.54]** | -0.51 [-3.87, 2.85] | 1.19 [-3.55, 5.88] |
| -0.02 [-1.92, 1.86] | -0.80 [-4.05, 2.47] | 0.05 [-1.85, 1.94] | **OLA** | -0.85 [-4.21, 2.50] | 0.84 [-3.88, 5.54] |
| 1.16 [-0.55, 2.87] | 0.38 [-2.77, 3.52] | 1.23 [-0.49, 2.96] | 1.18 [-1.35, 3.73] | **QUE** | 1.70 [-4.07, 7.46] |
| -1.51 [-4.99, 1.98] | -2.29 [-6.68, 2.11] | -1.43 [-4.92, 2.06] | -1.48 [-5.43, 2.48] | -2.66 [-6.56, 1.24] | **LUR** |

Note: Low-Density Lipoprotein results are on the top-right and Glucose results are on the bottom-left. Numbers represent the mean change [95% credible interval]. In the top-right comparisons, the row treatment is the reference category. In the bottom-left, the column treatment is the reference category.

Abbreviations: PLO – Placebo; ARI – Aripiprazole; CAR – Cariprazine; LUR – Lurasidone; OLA – Olanzapine; QUE – Quetiapine

**Appendix Table 5d. Fixed effect model for Change from Baseline in Prolactin**

| **PLO** |  |  |  |  |
| --- | --- | --- | --- | --- |
| 0.34 [-1.47, 2.15] | **ARI** |  |  |  |
| **2.22 [1.17, 3.27]** | 1.89 [-0.20, 3.98] | **CAR** |  |  |
| 0.91 [-1.36, 3.18] | 0.57 [-2.34, 3.49] | -1.32 [-3.82, 1.19] | **QUE** |  |
| **7.26 [2.20, 12.30]** | **6.92 [1.54, 12.29]** | 5.03 [-0.14, 10.20] | **6.35 [0.80, 11.87]** | **LUR** |

Note: Prolactin results are on the bottom-left. Numbers represent the mean change from baseline [95% credible interval]. In the top-right comparisons, the row treatment is the reference category. In the bottom-left, the column treatment is the reference category.

Abbreviations: PLO – Placebo; ARI – Aripiprazole; CAR – Cariprazine; LUR – Lurasidone; QUE – Quetiapine

**Appendix Table 6a. Fixed effect model for Odds Ratios for Somnolence and Switch to Mania**

| **PLO** | 2.03 [1.00, 3.75] | 1.93 [0.87, 3.96] | **2.87 [2.04, 3.95]** | **4.87 [3.62, 6.53]** | **5.06 [2.67, 9.20]** | 1.55 [0.57, 3.64] |
| --- | --- | --- | --- | --- | --- | --- |
| 2.15 [0.97, 4.25] | **ARI** | 1.07 [0.35, 2.58] | 1.58 [0.70, 3.05] | **2.69 [1.21, 5.14]** | **2.80 [1.02, 6.25]** | 0.85 [0.24, 2.30] |
| 0.93 [0.49, 1.63] | 0.50 [0.17, 1.14] | **CAR** | 1.72 [0.67, 3.50] | **2.93 [1.16, 5.86]** | 3.04 [1.00, 7.12] | 0.93 [0.24, 2.56] |
| 0.80 [0.41, 1.39] | **0.43 [0.15, 0.98]** | 0.95 [0.37, 2.00] | **OLA** | **1.75 [1.09, 2.66]** | 1.82 [0.86, 3.51] | 0.56 [0.19, 1.36] |
| **0.60 [0.38, 0.89]** | 0.32 [0.12, 0.67] | 0.70 [0.31, 1.35] | 0.82 [0.37, 1.59] | **QUE** | 1.06 [0.51, 2.02] | **0.33 [0.11, 0.79]** |
| NA | NA | NA | NA | NA | **ZIP** | **0.34 [0.10, 0.89]** |
| 2.32 [0.47, 7.97] | 1.24 [0.19, 4.73] | 2.73 [0.46, 9.95] | 3.17 [0.53, 11.72] | 4.07 [0.75, 14.64] | NA | **LUR** |

Note: Somnolence results are on the top-right and Switch to Mania results are on bottom-left. Numbers represent the mean change from baseline or odds ratio [95% credible interval]. In the top-right comparisons, the row treatment is the reference category.

Abbreviations: PLO – Placebo; ARI – Aripiprazole; CAR – Cariprazine; LUR – Lurasidone; OLA – Olanzapine; QUE – Quetiapine; ZIP – Ziprasidone

**Appendix Table 6b. – Fixed effect model for Odds Ratios for Extrapyramidal Symptoms and Akathisia**

| **PLO** | **1.94 [1.12, 3.18]** | **2.24 [1.40, 3.50]** | **2.66 [1.68, 4.11]** | **4.08 [1.22, 11.69]** |
| --- | --- | --- | --- | --- |
| **8.92 [4.84, 15.80]** | **ARI** | 1.24 [0.58, 2.34] | 1.48 [0.70, 2.77] | 2.27 [0.57, 6.95] |
| **3.54 [1.90, 6.35]** | **0.44 [0.17, 0.93]** | **CAR** | 1.25 [0.63, 2.27] | 1.93 [0.50, 5.79] |
| NA | NA | NA | **QUE** | 1.62 [0.43, 4.84] |
| **5.67 [1.72, 16.42]** | 0.70 [0.17, 2.16] | 1.76 [0.42, 5.48] | NA | **LUR** |

Note: Extrapyramidal Symptom results are on the top-right. Akathisia results are on bottom left. Results give the odds ratio [95% credible interval]. In the top-right comparisons, the row treatment is the reference treatment. In the bottom-left, the column treatment is the reference category.

Abbreviations: PLO – Placebo; ARI – Aripiprazole; CAR – Cariprazine; LUR – Lurasidone; QUE – Quetiapine

**Appendix Table 7a. – GRADE assessment of Continuous Outcomes**

| Comparison | Direct evidence | | | Network meta-analysis | |
| --- | --- | --- | --- | --- | --- |
|  | Number of studies | Mean difference (95% confidence interval) | Quality of evidence | Mean difference (95% confidence interval) | Quality of evidence |
| **Change from Baseline in MADRS** | | | | | |
| Lurasidone vs placebo | 1 | -4.7 [-6.33, -3.07] | High | -4.71 [-6.98, -2.41] | High |
| Lurasidone vs Aripiprazole | 0 |  |  | -3.63 [-6.78, -0.50] | Moderate^#^ |
| Lurasidone vs Cariprazine | 0 |  |  | -2.42 [-5.01, 0.14] | Low^#%^ |
| Lurasidone vs Olanzapine | 0 |  |  | -0.13 [-2.82, 2.50] | Low^#%^ |
| Lurasidone vs Quetiapine | 0 |  |  | 0.10 [-2.44, 2.61] | Low^#%^ |
| Lurasidone vs Ziprasidone | 0 |  |  | -3.36 [-6.38, -0.39] | Moderate^#^ |
| Ziprasidone vs placebo | 2 | -1.25 [-2.84, 0.33] | Low*^%^ | -1.34 [-3.29, 0.67] | Low*^%^ |
| Ziprasidone vs Aripiprazole | 0 |  |  | -0.27 [-3.24, 2.71] | Very low*^#%^ |
| Ziprasidone vs Cariprazine | 0 |  |  | 0.94 [-1.34, 3.27] | Very low*^#%^ |
| Ziprasidone vs Olanzapine | 0 |  |  | 3.23 [0.83, 5.66] | Very low*^#%^ |
| Ziprasidone vs Quetiapine | 0 |  |  | 3.46 [1.24, 5.76] | Low*^#^ |
| Quetiapine vs placebo | 6 | -4.83 [-5.66, -3.99] | High | -4.80 [-5.93, -3.72] | High |
| Quetiapine vs Aripiprazole | 0 |  |  | ‐3.72 [‐6.21, ‐1.28] | Moderate^#^ |
| Quetiapine vs Cariprazine | 0 |  |  | ‐2.52 [‐4.11, ‐0.92] | Moderate^#^ |
| Quetiapine vs Olanzapine | 0 |  |  | -0.23 [-2.05, 1.53] | Low^#%^ |
| Olanzapine vs placebo | 3 | -4.65 [-5.79, -3.50] | High | -4.57 [-5.92, -3.16] | High |
| Olanzapine vs Aripiprazole | 0 |  |  | ‐3.49 [‐6.07, ‐0.92] | Moderate^#^ |
| Olanzapine vs Cariprazine | 0 |  |  | ‐2.29 [‐4.09, ‐0.46] | Moderate^#^ |
| Cariprazine vs placebo | 4 | -2.36 [-3.17, -1.55] | High | -2.29 [-3.47, -1.09] | High |
| Cariprazine vs Aripiprazole | 0 |  |  | -1.21 [-3.70, 1.29] | Low^#%^ |
| Aripiprazole vs placebo | 2 | -1.07 [-3.03, 0.89] | Low*^%^ | -1.08 [-3.26, 1.10] | Low*^%^ |
| **Change from Baseline in CGI-BP-S-depression** | | | | | |
| Lurasidone vs placebo | 1 | -0.63 [-0.83, -0.43] | High | ‐0.63 [‐1.12, ‐0.14] | High |
| Lurasidone vs Aripiprazole | 0 |  |  | -0.42 [-1.06, 0.23] | Low^#%^ |
| Lurasidone vs Olanzapine | 0 |  |  | -0.31 [-0.95, 0.33] | Low^#%^ |
| Olanzapine vs placebo | 2 | -0.32 [-0.51, -0.13] | High | -0.32 [-0.72, 0.08] | Moderate^%^ |
| Olanzapine vs Aripiprazole | 0 |  |  | -0.11 [-0.68, 0.47] | Low^#%^ |
| Aripiprazole vs placebo | 2 | -0.21 [-0.43, 0.01] | Low*^%^ | -0.21 [-0.63, 0.20] | Low*^%^ |
| **Change from Baseline in CGI-BP-S-overall** | | | | | |
| Lurasidone vs placebo | 1 | -0.52 [-0.72, -0.32] | Moderate^ | ‐0.63 [‐0.87,‐0.39] | Moderate^ |
| Lurasidone vs Cariprazine | 0 |  |  | ‐0.38 [‐0.66,‐0.10] | Moderate^#^ |
| Lurasidone vs Olanzapine | 0 |  |  | -0.04 [-1.41, 1.46] | Low^#%^ |
| Lurasidone vs Quetiapine | 0 |  |  | -0.08 [-0.36, 0.19] | Low^#%^ |
| Lurasidone vs Ziprasidone | 0 |  |  | ‐0.58 [‐0.91,‐0.26] | Moderate^#^ |
| Ziprasidone vs placebo | 2 | -0.04 [-0.23, 0.15] | Very low*^%^^ | -0.05 [-0.26, 0.17] | Very low*^#%^^ |
| Ziprasidone vs Cariprazine | 0 |  |  | 0.20 [-0.05, 0.46] | Very low*^#%^ |
| Ziprasidone vs Olanzapine | 0 |  |  | 0.54 [-0.83, 2.03] | Very low*^#%^ |
| Ziprasidone vs Quetiapine | 0 |  |  | 0.50 [0.24, 0.76] | Low*^#^ |
| Quetiapine vs placebo | 5 | -0.55 [-0.65, -0.44] | Moderate^ | ‐0.55 [‐0.68 ‐0.41] | Moderate^ |
| Quetiapine vs Cariprazine | 0 |  |  | ‐0.30 [‐0.49,‐0.11] | Moderate^#^ |
| Quetiapine vs Olanzapine | 0 |  |  | 0.04 [-1.31, 1.51] | Low*^#^ |
| Olanzapine vs placebo | 1 | -0.62 [-2.09, 0.85] | Low^%^^ | -0.59 [-2.06, 0.76] | Low^%^^ |
| Olanzapine vs Cariprazine | 0 |  |  | -0.34 [-1.82, 1.01] | Low^#%^ |
| Cariprazine vs placebo | 4 | -0.25 [-0.35, -0.16] | Moderate^ | ‐0.25 [‐0.38,‐0.11] | Moderate^ |
| **Change from Baseline in Weight** | | | | | |
| Lurasidone vs placebo | 1 | 0.34 [0.04, 0.65] | High | 0.34 [-0.22, 0.89] | Moderate^%^ |
| Lurasidone vs Aripiprazole | 0 |  |  | 0.14 [-0.85, 1.11] | Low^#%^ |
| Lurasidone vs Cariprazine | 0 |  |  | -0.31 [-0.95, 0.33] | Low^#%^ |
| Lurasidone vs Olanzapine | 0 |  |  | ‐2.54 [‐3.28, ‐1.81] | Moderate^%^ |
| Lurasidone vs Quetiapine | 0 |  |  | ‐0.83 [‐1.48, ‐0.18] | Moderate^%^ |
| Quetiapine vs placebo | 5 | 1.16 [0.95, 1.38] | Low^%^ | 1.17 [0.84, 1.49] | Moderate^%^ |
| Quetiapine vs Aripiprazole | 0 |  |  | 0.96 [0.10, 1.83] | Low^#%^ |
| Quetiapine vs Cariprazine | 0 |  |  | 0.52 [0.07, 0.96] | Low^#%^ |
| Quetiapine vs Olanzapine | 0 |  |  | ‐1.71 [‐2.29, ‐1.13] | Moderate^#^ |
| Olanzapine vs placebo | 3 | 2.90 [2.57, 3.24] | High | 2.88 [2.40, 3.36] | High |
| Olanzapine vs Aripiprazole | 0 |  |  | 2.68 [1.76, 3.61] | Moderate^#^ |
| Olanzapine vs Cariprazine | 0 |  |  | 2.24 [1.66, 2.80] | Moderate^#^ |
| Cariprazine vs placebo | 4 | 0.65 [0.47, 0.83] | High | 0.65 [0.34, 0.96] | High |
| Cariprazine vs Aripiprazole | 0 |  |  | 0.44 [-0.42, 1.30] | Low^#%^ |
| Aripiprazole vs placebo | 2 | 0.19 [-0.53, 0.90] | Low*^%^ | 0.20 [-0.59, 1.00] | Low*^%^ |
| **Change from Baseline in Triglycerides** | | | | | |
| Lurasidone vs placebo | 1 | -3.01 [-12.93, 6.90] | Low^%^^ | -3.05 [-15.37, 9.55] | Low^^%^ |
| Lurasidone vs Aripiprazole | 0 |  |  | -4.03 [-21.02, 12.83] | Low^#%^ |
| Lurasidone vs Cariprazine | 0 |  |  | -4.40 [-17.54, 8.47] | Low^#%^ |
| Lurasidone vs Olanzapine | 0 |  |  | -4.89 [-18.60, 8.37] | Low^#%^ |
| Lurasidone vs Quetiapine | 0 |  |  | -14.15 [-32.40, 4.08] | Low^#%^ |
| Quetiapine vs placebo | 3 | 10.70 [-0.30, 21.71] | Low^%^^ | 11.10 [-2.75, 24.86] | Low^%^^ |
| Quetiapine vs Aripiprazole | 0 |  |  | 10.12 [-7.63, 28.42] | Low^%#^ |
| Quetiapine vs Cariprazine | 0 |  |  | 9.75 [-4.84, 24.02] | Low^%#^ |
| Quetiapine vs Olanzapine | 0 |  |  | 9.25 [-5.75, 23.54] | Low^%#^ |
| Olanzapine vs placebo | 2 | 0.72 [0.21, 1.22] | Low^%^^ | 1.85 [-1.88, 8.64] | Low^%^^ |
| Olanzapine vs Aripiprazole | 0 |  |  | 0.87 [-11.26, 13.80] | Low^#%^ |
| Olanzapine vs Cariprazine | 0 |  |  | 0.50 [-5.46, 6.84] | Low^#%^ |
| Cariprazine vs placebo | 4 | 0.20 [-0.01, 0.40] | Low^%^^ | 1.35 [-1.27, 6.50] | Low^%^^ |
| Cariprazine vs Aripiprazole | 0 |  |  | 0.37 [-11.45, 12.83] | Low^#%^ |
| Aripiprazole vs placebo | 2 | 1.03 [-10.19, 12.26] | Very low*^%^^ | 0.98 [-10.48, 12.47] | Very low*^%^^ |
| **Change from Baseline in Total Cholesterol** | | | | | |
| Lurasidone vs placebo | 1 | 1.74 [-2.74, 6.22] | Moderate^%^ | 1.72 [-6.56, 9.94] | Moderate^%^ |
| Lurasidone vs Aripiprazole | 0 |  |  | 1.22 [-9.02, 11.51] | Low^#%^ |
| Lurasidone vs Cariprazine | 0 |  |  | 3.77 [-5.24, 12.88] | Low^#%^ |
| Lurasidone vs Olanzapine | 0 |  |  | -5.34 [-14.99, 4.03] | Low^#%^ |
| Lurasidone vs Quetiapine | 0 |  |  | 1.21 [-8.64, 10.98] | Low^#%^ |
| Quetiapine vs placebo | 3 | 0.41 [-2.83, 3.66] | Moderate^%^ | 0.50 [-4.86, 5.88] | Moderate^%^ |
| Quetiapine vs Aripiprazole | 0 |  |  | 0.00 [-8.13, 8.18] | Low^#%^ |
| Quetiapine vs Cariprazine | 0 |  |  | 2.55 [-3.96, 9.20] | Low^#%^ |
| Quetiapine vs Olanzapine | 0 |  |  | -6.55 [-13.85, 0.46] | Low^#%^ |
| Olanzapine vs placebo | 3 | 1.29 [0.63, 1.95] | Moderate^%^ | 7.06 [2.47, 12.00] | High |
| Olanzapine vs Aripiprazole | 0 |  |  | 6.55 [-1.05, 14.47] | Low^#%^ |
| Olanzapine vs Cariprazine | 0 |  |  | 9.11 [3.22, 15.46] | Moderate^%^ |
| Cariprazine vs placebo | 4 | 0.02 [-0.16, 0.19] | Moderate^%^ | -2.05 [-5.90, 1.67] | Moderate^%^ |
| Cariprazine vs Aripiprazole | 0 |  |  | -2.55 [-9.87, 4.56] | Low^#%^ |
| Aripiprazole vs placebo | 2 | 0.30 [-3.18, 3.78] | Low*^%^ | 0.50 [-5.64, 6.60] | Low*^%^ |
| **Change from Baseline in Low-Density Lipoprotein Cholesterol** | | | | | |
| Lurasidone vs placebo | 1 | 0.98 [-2.92, 7.52] | Moderate^%^ | 1.18 [-3.86, 6.23] | Moderate^%^ |
| Lurasidone vs Aripiprazole | 0 |  |  | 1.67 [-4.52, 7.81] | Low^#%^ |
| Lurasidone vs Cariprazine | 0 |  |  | 1.84 [-3.28, 7.39] | Low^#%^ |
| Lurasidone vs Olanzapine | 0 |  |  | 0.75 [-4.51, 6.04] | Low^#%^ |
| Lurasidone vs Quetiapine | 0 |  |  | 1.76 [-4.42, 7.84] | Low^#%^ |
| Quetiapine vs placebo | 3 | -0.60 [-3.43, 2.23] | Moderate^%^ | -0.59 [-4.23, 3.00] | Moderate^%^ |
| Quetiapine vs Aripiprazole | 0 |  |  | -0.09 [-5.29, 4.95] | Low^#%^ |
| Quetiapine vs Cariprazine | 0 |  |  | 0.08 [-3.80, 4.44] | Low^#%^ |
| Quetiapine vs Olanzapine | 0 |  |  | -1.01 [-5.01, 2.96] | Low^#%^ |
| Olanzapine vs placebo | 2 | 0.27 [0.14, 0.39] | High | 0.42 [-1.23, 2.16] | Moderate^%^ |
| Olanzapine vs Aripiprazole | 0 |  |  | 0.92 [-3.03, 4.83] | Low^#%^ |
| Olanzapine vs Cariprazine | 0 |  |  | 1.09 [-0.48, 4.43] | Low^#%^ |
| Cariprazine vs placebo | 4 | -0.07 [-0.21, 0.06] | Moderate^%^ | -0.67 [-3.23, 0.42] | Low^#%^ |
| Cariprazine vs Aripiprazole | 0 |  |  | -0.17 [-4.61, 3.60] | Low^#%^ |
| Aripiprazole vs placebo | 2 | -0.50 [-3.73, 2.72] | Low*^%^ | -0.50 [-4.09, 3.14] | Low*^%^ |
| **Change from Baseline in Glucose** | | | | | |
| Lurasidone vs placebo | 1 | -1.39 [-4.21, 1.43] | Moderate^%^ | -1.45 [-5.50, 2.64] | Moderate^%^ |
| Lurasidone vs Aripiprazole | 0 |  |  | -2.35 [-7.61, 2.73] | Low^#%^ |
| Lurasidone vs Cariprazine | 0 |  |  | -1.52 [-5.85, 2.77] | Low^#%^ |
| Lurasidone vs Olanzapine | 0 |  |  | -1.11 [-5.85, 3.94] | Low^#%^ |
| Lurasidone vs Quetiapine | 0 |  |  | -2.60 [-7.12, 1.92] | Low^#%^ |
| Quetiapine vs placebo | 6 | 1.17 [-0.27, 2.61] | Moderate^%^ | 1.15 [-0.82, 3.12] | Moderate^%^ |
| Quetiapine vs Aripiprazole | 0 |  |  | 0.25 [-3.56, 3.91] | Low^#%^ |
| Quetiapine vs Cariprazine | 0 |  |  | 1.08 [-1.46, 3.44] | Low^#%^ |
| Quetiapine vs Olanzapine | 0 |  |  | 1.50 [-1.72, 4.94] | Low^#%^ |
| Olanzapine vs placebo | 2 | -0.04 [-1.93, 1.85] | Moderate^%^ | -0.34 [-3.18, 2.17] | Moderate^%^ |
| Olanzapine vs Aripiprazole | 0 |  |  | -1.25 [-5.62, 2.71] | Low^#%^ |
| Olanzapine vs Cariprazine | 0 |  |  | -0.42 [-3.78, 2.39] | Low^#%^ |
| Cariprazine vs placebo | 4 | -0.08 [-0.25, 0.10] | Moderate^%^ | 0.07 [-1.31, 1.70] | Moderate^%^ |
| Cariprazine vs Aripiprazole | 0 |  |  | -0.83 [-4.29, 2.59] | Low^#%^ |
| Aripiprazole vs placebo | 2 | 0.77 [-1.87, 3.41] | Low*^%^ | 0.90 [-2.17, 4.12] | Low*^%^ |
| **Change from Baseline in Prolactin** | | | | | |
| Lurasidone vs placebo | 1 | 7.26 [3.36, 11.16] | High | 7.20 [2.06, 12.33] | High |
| Lurasidone vs Aripiprazole | 0 |  |  | 6.83 [1.25, 12.37] | Moderate^%^ |
| Lurasidone vs Cariprazine | 0 |  |  | 4.98 [-0.36, 10.34] | Low^#%^ |
| Lurasidone vs Quetiapine | 0 |  |  | 6.16 [0.41, 11.79] | Low^#%^ |
| Quetiapine vs placebo | 2 | 1.02 [-0.81, 2.85] | Moderate^%^ | 1.04 [-1.42, 3.55] | Moderate^%^ |
| Quetiapine vs Aripiprazole | 0 |  |  | 0.67 [-2.57, 3.91] | Low^#%^ |
| Quetiapine vs Cariprazine | 0 |  |  | -1.18 [-4.02, 1.70] | Low^#%^ |
| Cariprazine vs placebo | 3 | 2.21 [1.36, 3.05] | High | 2.22 [0.89, 3.57] | Moderate^%^ |
| Cariprazine vs Aripiprazole | 0 |  |  | 1.85 [-0.60, 4.35] | Low^#%^ |
| Aripiprazole vs placebo | 2 | 0.34 [-1.47, 2.14] | Low*^%^ | 0.37 [-1.72, 2.42] | Low*^%^ |

Factors downgrading any specific evidence: * Limitations (risk of bias), $ Inconsistency of results, # indirectness of results, % Imprecision, ^ Publications bias

**Appendix Table 7b. – GRADE assessment of Dichotomous Outcomes**

| Comparison | Direct evidence | | | | Network meta-analysis | |
| --- | --- | --- | --- | --- | --- | --- |
|  | Number of studies | | Odds ratio (95% confidence interval) | Quality of evidence | Odds ratio (95% confidence interval) | Quality of evidence |
| **Odds Ratio for Response** | | | | | | |
| Lurasidone vs placebo | 1 | 2.50 [1.81, 3.45] | | Moderate^ | 2.58 [1.67, 3.81] | Moderate^ |
| Lurasidone vs Aripiprazole | 0 |  | |  | 2.38 [1.38, 3.85] | Moderate^#^ |
| Lurasidone vs Cariprazine | 0 |  | |  | 1.78 [1.08, 2.77] | Moderate^#^ |
| Lurasidone vs Olanzapine | 0 |  | |  | 1.68 [0.99, 2.65] | Low^#%^ |
| Lurasidone vs Quetiapine | 0 |  | |  | 1.25 [0.78, 1.90] | Low^#%^ |
| Lurasidone vs Ziprasidone | 0 |  | |  | 2.47 [1.41, 3.98] | Moderate^#^ |
| Ziprasidone vs placebo | 2 | 1.06 [0.81, 1.38] | | Very low^*%^^ | 1.07 [0.77, 1.46] | Very Low*^%^^ |
| Ziprasidone vs Aripiprazole | 0 |  | |  | 0.99 [0.61, 1.50] | Very Low*^#%^ |
| Ziprasidone vs Cariprazine | 0 |  | |  | 0.74 [0.50, 1.08] | Very Low*^#%^ |
| Ziprasidone vs Olanzapine | 0 |  | |  | 0.70 [0.45, 1.03] | Very Low*^#%^ |
| Ziprasidone vs Quetiapine | 0 |  | |  | 0.52 [0.35, 0.74] | Low*^#^ |
| Quetiapine vs placebo | 6 | 2.03 [1.76, 2.34] | | Moderate^ | 2.09 [1.74, 2.49] | Moderate^ |
| Quetiapine vs Aripiprazole | 0 |  | |  | 1.92 [1.32, 2.71] | Moderate^#^ |
| Quetiapine vs Cariprazine | 0 |  | |  | 1.44 [1.08, 1.91] | Moderate^#^ |
| Quetiapine vs Olanzapine | 0 |  | |  | 1.36 [0.98, 1.84] | Low^#%^ |
| Olanzapine vs placebo | 3 | 1.58 [1.15, 2.17] | | Moderate^ | 1.56 [1.20, 2.01] | Moderate^ |
| Olanzapine vs Aripiprazole | 0 |  | |  | 1.44 [0.94, 2.14] | Low^#%^ |
| Olanzapine vs Cariprazine | 0 |  | |  | 1.08 [0.76, 1.51] | Low^#%^ |
| Cariprazine vs placebo | 4 | 1.47 [1.25, 1.75] | | Moderate^ | 1.47 [1.17, 1.82] | Moderate^ |
| Cariprazine vs Aripiprazole | 0 |  | |  | 1.35 [0.90, 1.95] | Low^#%^ |
| Aripiprazole vs placebo | 2 | 1.09 [0.81, 1.47] | | Very Low*^%^^ | 1.11 [0.80, 1.51] | Very Low*^%^^ |
| **Odds Ratios for Remission (MADRS≤ 12)** | | | | | | |
| Lurasidone vs placebo | 1 | 2.11 [1.51, 2.95] | | High | 2.20 [1.35, 3.40] | High |
| Lurasidone vs Olanzapine | 0 |  | |  | 1.56 [0.86, 2.53] | Low^#%^ |
| Lurasidone vs Quetiapine | 0 |  | |  | 1.09 [0.64, 1.73] | Low^#%^ |
| Lurasidone vs Ziprasidone | 0 |  | |  | 2.19 [1.21, 3.64] | Moderate^#^ |
| Ziprasidone vs placebo | 2 | 1.01 [0.79, 1.29] | | Low*^%^ | 1.03 [0.75, 1.39] | Low*^%^ |
| Ziprasidone vs Olanzapine | 0 |  | |  | 0.73 [0.47, 1.06] | Very low*^#%^ |
| Ziprasidone vs Quetiapine | 0 |  | |  | 0.51 [0.35, 0.72] | Low*^#^ |
| Quetiapine vs placebo | 6 | 2.01 [1.74, 2.32] | | High | 2.04 [1.70, 2.44] | High |
| Quetiapine vs Olanzapine | 0 |  | |  | 1.45 [1.02, 1.99] | Moderate^%^ |
| Olanzapine vs placebo | 3 | 1.47 [1.00, 2.14] | | High | 1.44 [1.10, 1.88] | High |
| **Odds Ratios for Remission (MADRS≤ 10)** | | | | | | |
| Lurasidone vs placebo | 1 | 2.05 [1.44, 2.92] | | High | 2.16 [1.14, 3.76] | High |
| Lurasidone vs Cariprazine | 0 |  | |  | 1.39 [0.66, 2.60] | Low^#%^ |
| Cariprazine vs placebo | 3 | 1.58 [1.30, 1.93] | | High | 1.61 [1.12 ,2.23] | High |
| **Odds Ratios for All-Cause Discontinuation** | | | | | | |
| Lurasidone vs placebo | 1 | 1.05 [0.75, 1.49] | | Moderate^%^ | 1.10 [0.61, 1.83] | High |
| Lurasidone vs Aripiprazole | 0 |  | |  | 0.68 [0.32, 1.28] | Low^#%^ |
| Lurasidone vs Cariprazine | 0 |  | |  | 1.07 [0.54, 1.90] | Low^#%^ |
| Lurasidone vs Olanzapine | 0 |  | |  | 1.62 [0.79, 2.95] | Low^#%^ |
| Lurasidone vs Quetiapine | 0 |  | |  | 1.12 [0.59, 1.95] | Low^#%^ |
| Lurasidone vs Ziprasidone | 0 |  | |  | 0.83 [0.40, 1.54] | Low^#%^ |
| Ziprasidone vs placebo | 2 | 1.35 [1.05, 1.73] | | Moderate* | 1.38 [0.92, 1.98] | Very low*^#%^ |
| Ziprasidone vs Aripiprazole | 0 |  | |  | 0.86 [0.47, 1.45] | Very low*^#%^ |
| Ziprasidone vs Cariprazine | 0 |  | |  | 1.34 [0.79, 2.11] | Very low*^#%^ |
| Ziprasidone vs Olanzapine | 0 |  | |  | 2.03 [1.15, 3.30] | Low*^#^ |
| Ziprasidone vs Quetiapine | 0 |  | |  | 1.41 [0.88, 2.15] | Very low*^#%^ |
| Quetiapine vs placebo | 6 | 0.98 [0.81, 1.20] | | Moderate^%^ | 0.99 [0.78, 1.24] | Moderate^%^ |
| Quetiapine vs Aripiprazole | 0 |  | |  | 0.62 [0.37, 0.96] | Moderate^#^ |
| Quetiapine vs Cariprazine | 0 |  | |  | 0.96 [0.64, 1.37] | Low^#%^ |
| Quetiapine vs Olanzapine | 0 |  | |  | 1.46 [0.93, 2.18] | Low^#%^ |
| Olanzapine vs placebo | 3 | 0.68 [0.54, 0.87] | | High | 0.70 [0.48, 1.00] | Moderate^%^ |
| Olanzapine vs Aripiprazole | 0 |  | |  | 0.44 [0.24, 0.73] | Moderate^#^ |
| Olanzapine vs Cariprazine | 0 |  | |  | 0.68 [0.42, 1.06] | Low^#%^ |
| Cariprazine vs placebo | 4 | 1.04 [0.85, 1.27] | | Moderate^%^ | 1.05 [0.77, 1.41] | Moderate^%^ |
| Cariprazine vs Aripiprazole | 0 |  | |  | 0.66 [0.38, 1.06] | Low^#%^ |
| Aripiprazole vs placebo | 2 | 1.64 [1.22, 2.20] | | Moderate* | 1.68 [1.09, 2.48] | Moderate* |
| **Odds Ratios for Discontinuation Due to Adverse Events** | | | | | | |
| Lurasidone vs placebo | 1 | 0.97 [0.52, 1.79] | | Moderate^%^ | 1.12 [0.36, 2.76] | Moderate^%^ |
| Lurasidone vs Aripiprazole | 0 |  | |  | 0.52 [0.12, 1.50] | Low^#%^ |
| Lurasidone vs Cariprazine | 0 |  | |  | 0.81 [0.21, 2.23] | Low^#%^ |
| Lurasidone vs Olanzapine | 0 |  | |  | 0.88 [0.22, 2.50] | Low^#%^ |
| Lurasidone vs Quetiapine | 0 |  | |  | 0.48 [0.14, 1.24] | Low^#%^ |
| Lurasidone vs Ziprasidone | 0 |  | |  | 0.81 [0.20, 2.27] | Low^#%^ |
| Ziprasidone vs placebo | 2 | 1.45 [1.00, 2.10] | | Moderate* | 1.54 [0.76, 2.80] | Low*^%^ |
| Ziprasidone vs Aripiprazole | 0 |  | |  | 0.72 [0.23, 1.70] | Very low*^#%^ |
| Ziprasidone vs Cariprazine | 0 |  | |  | 1.12 [0.41, 2.37] | Very low*^#%^ |
| Ziprasidone vs Olanzapine | 0 |  | |  | 1.21 [0.43, 2.82] | Very low*^#%^ |
| Ziprasidone vs Quetiapine | 0 |  | |  | 0.66 [0.27, 1.32] | Very low*^#%^ |
| Quetiapine vs placebo | 6 | 2.23 [1.45, 3.43] | | High | 2.46 [1.57, 3.75] | High |
| Quetiapine vs Aripiprazole | 0 |  | |  | 1.15 [0.44, 2.49] | Low^#%^ |
| Quetiapine vs Cariprazine | 0 |  | |  | 1.79 [0.80, 3.42] | Low^#%^ |
| Quetiapine vs Olanzapine | 0 |  | |  | 1.93 [0.82, 4.09] | Low^#%^ |
| Olanzapine vs placebo | 3 | 1.38 [0.77, 2.48] | | Moderate^%^ | 1.43 [0.68, 2.58] | Moderate^%^ |
| Olanzapine vs Aripiprazole | 0 |  | |  | 0.67 [0.21, 1.57] | Low^#%^ |
| Olanzapine vs Cariprazine | 0 |  | |  | 1.04 [0.37, 2.23] | Low^#%^ |
| Cariprazine vs placebo | 4 | 1.25 [0.89, 1.76] | | Moderate^%^ | 1.50 [0.82, 2.64] | Moderate^%^ |
| Cariprazine vs Aripiprazole | 0 |  | |  | 0.70 [0.25, 1.64] | Low^#%^ |
| Aripiprazole vs placebo | 2 | 2.28 [1.37, 3.80] | | Moderate* | 2.47 [1.10, 4.90] | Moderate* |
| **Odds Ratios for Discontinuation Due Lack of Efficacy** | | | | | | |
| Lurasidone vs placebo | 1 | 0.63 [0.25, 1.56] | | Moderate^%^ | 0.77 [0.18, 2.17] | Moderate^%^ |
| Lurasidone vs Aripiprazole | 0 |  | |  | 1.58 [0.21, 5.73] | Low^#%^ |
| Lurasidone vs Cariprazine | 0 |  | |  | 1.84 [0.31, 6.17] | Low^#%^ |
| Lurasidone vs Olanzapine | 0 |  | |  | 2.32 [0.35, 8.31] | Low^#%^ |
| Lurasidone vs Quetiapine | 0 |  | |  | 3.72 [0.71, 11.61] | Low^#%^ |
| Lurasidone vs Ziprasidone | 0 |  | |  | 0.57 [0.07, 2.06] | Low^#%^ |
| Ziprasidone vs placebo | 2 | 1.57 [0.78, 3.14] | | Low*^%^ | 1.84 [0.54, 4.88] | Low*^%^ |
| Ziprasidone vs Aripiprazole | 0 |  | |  | 3.79 [0.62, 13.01] | Very low*^#%^ |
| Ziprasidone vs Cariprazine | 0 |  | |  | 4.41 [0.89, 14.22] | Very low*^#%^ |
| Ziprasidone vs Olanzapine | 0 |  | |  | 5.55 [1.03, 19.22] | Low*^#^ |
| Ziprasidone vs Quetiapine | 0 |  | |  | 8.91 [2.12, 26.64] | Low*^#^ |
| Quetiapine vs placebo | 6 | 0.25 [0.15, 0.40] | | High | 0.23 [0.12, 0.40] | High |
| Quetiapine vs Aripiprazole | 0 |  | |  | 0.47 [0.11, 1.30] | Low^#%^ |
| Quetiapine vs Cariprazine | 0 |  | |  | 0.54 [0.17, 1.33] | Low^#%^ |
| Quetiapine vs Olanzapine | 0 |  | |  | 0.68 [0.19, 1.84] | Low^#%^ |
| Olanzapine vs placebo | 2 | 0.38 [0.17, 0.87] | | High | 0.42 [0.14, 0.93] | High |
| Olanzapine vs Aripiprazole | 0 |  | |  | 0.86 [0.16, 2.68] | Low^#%^ |
| Olanzapine vs Cariprazine | 0 |  | |  | 1.01 [0.23, 2.83] | Low^#%^ |
| Cariprazine vs placebo | 4 | 0.56 [0.32, 0.98] | | High | 0.50 [0.20, 1.04] | Moderate^%^ |
| Cariprazine vs Aripiprazole | 0 |  | |  | 1.02 [0.21, 3.06] | Low^#%^ |
| Aripiprazole vs placebo | 2 | 0.58 [0.23, 1.45] | | Low*^%^ | 0.65 [0.19, 1.60] | Low*^%^ |
| **Odds Ratios of ≥7% Weight Gain** | | | | | | |
| Lurasidone vs placebo | 1 | 3.24 [0.55, 19.24] | | Low^^%^ | 19.08 [0.66, 108.10] | Low^^%^ |
| Lurasidone vs Aripiprazole | 0 |  | |  | 14.68 [0.35, 83.24] | Low^#%^ |
| Lurasidone vs Cariprazine | 0 |  | |  | 6.85 [0.16, 39.89] | Low^#%^ |
| Lurasidone vs Olanzapine | 0 |  | |  | 0.43 [0.01, 2.56] | Low^#%^ |
| Lurasidone vs Quetiapine | 0 |  | |  | 5.98 [0.18, 33.08] | Low^#%^ |
| Quetiapine vs placebo | 6 | 2.85 [1.94, 4.19] | | Moderate^ | 3.46 [1.91, 5.92] | Moderate^ |
| Quetiapine vs Aripiprazole | 0 |  | |  | 2.64 [0.74, 6.90] | Low^#%^ |
| Quetiapine vs Cariprazine | 0 |  | |  | 1.26 [0.34, 3.13] | Low^#%^ |
| Quetiapine vs Olanzapine | 0 |  | |  | 0.08 [0.01, 0.25] | Moderate^#^ |
| Olanzapine vs placebo | 2 | 37.44 [11.83, 118.53] | | Moderate^ | 68.46 [15.56, 231.00] | Moderate^ |
| Olanzapine vs Aripiprazole | 0 |  | |  | 52.56 [7.42, 205.90] | Moderate^#^ |
| Olanzapine vs Cariprazine | 0 |  | |  | 24.93 [3.35, 95.57] | Moderate^#^ |
| Cariprazine vs placebo | 4 | 2.47 [1.13, 5.40] | | Moderate^ | 3.50 [1.26, 8.65] | Moderate^ |
| Cariprazine vs Aripiprazole | 0 |  | |  | 2.67 [0.56, 8.41] | Low^#%^ |
| Aripiprazole vs placebo | 2 | 1.47 [0.69, 3.15] | | Very Low*^%^^ | 1.67 [0.56, 3.92] | Very Low*^%^^ |
| **Odds Ratios for Somnolence** | | | | | | |
| Lurasidone vs placebo | 1 | 1.32 [0.64, 2.71] | | Moderate^%^ | 1.53 [0.57, 3.66] | Moderate^%^ |
| Lurasidone vs Aripiprazole | 0 |  | |  | 0.84 [0.24, 2.28] | Low^#%^ |
| Lurasidone vs Cariprazine | 0 |  | |  | 0.93 [0.23, 2.52] | Low^#%^ |
| Lurasidone vs Olanzapine | 0 |  | |  | 0.55 [0.18, 1.40] | Low^#%^ |
| Lurasidone vs Quetiapine | 0 |  | |  | 0.32 [0.11, 0.80] | Moderate^%^ |
| Lurasidone vs Ziprasidone | 0 |  | |  | 0.33 [0.09, 0.87] | Moderate^%^ |
| Ziprasidone vs placebo | 2 | 4.36 [2.64, 7.18] | | Moderate* | 5.05 [2.61, 9.25] | Moderate* |
| Ziprasidone vs Aripiprazole | 0 |  | |  | 2.79 [0.98, 6.44] | Very low*^#%^ |
| Ziprasidone vs Cariprazine | 0 |  | |  | 3.12 [0.99, 7.67] | Very low*^#%^ |
| Ziprasidone vs Olanzapine | 0 |  | |  | 1.81 [0.81, 3.57] | Very low*^#%^ |
| Ziprasidone vs Quetiapine | 0 |  | |  | 1.05 [0.50, 2.00] | Very low*^#%^ |
| Quetiapine vs placebo | 6 | 4.52 [3.45, 5.96] | | High | 4.90 [3.59, 6.56] | High |
| Quetiapine vs Aripiprazole | 0 |  | |  | 2.70 [1.21, 5.31] | Moderate^#^ |
| Quetiapine vs Cariprazine | 0 |  | |  | 3.03 [1.13, 6.47] | Moderate^#^ |
| Quetiapine vs Olanzapine | 0 |  | |  | 1.76 [1.06, 2.75] | Moderate^#^ |
| Olanzapine vs placebo | 2 | 2.81 [2.02, 3.91] | | High | 2.89 [1.96, 4.20] | High |
| Olanzapine vs Aripiprazole | 0 |  | |  | 1.59 [0.68, 3.16] | Low^#%^ |
| Olanzapine vs Cariprazine | 0 |  | |  | 1.79 [0.64, 3.85] | Low^#%^ |
| Cariprazine vs placebo | 2 | 1.64 [0.97, 2.77] | | Moderate^%^ | 1.90 [0.81, 4.05] | Moderate^%^ |
| Cariprazine vs Aripiprazole | 0 |  | |  | 1.04 [0.32, 2.61] | Low^#%^ |
| Aripiprazole vs placebo | 2 | 1.90 [0.99, 3.63] | | Low*^%^ | 2.04 [0.98, 3.79] | Low*^%^ |
| **Odds Ratios for Extrapyramidal Symptoms** | | | | | | |
| Lurasidone vs placebo | 1 | 3.00 [1.31, 6.85] | | Moderate^ | 4.12 [1.05, 12.76] | Moderate^ |
| Lurasidone vs Aripiprazole | 0 |  | |  | 2.33 [0.47 ,7.85] | Low^#%^ |
| Lurasidone vs Cariprazine | 0 |  | |  | 1.98 [0.43, 6.51] | Low^#%^ |
| Lurasidone vs Quetiapine | 0 |  | |  | 1.60 [0.35, 5.27] | Low^#%^ |
| Quetiapine vs placebo | 5 | 2.37 [1.68, 3.35] | | Moderate^ | 2.76 [1.61, 4.58] | Moderate^ |
| Quetiapine vs Aripiprazole | 0 |  | |  | 1.57 [0.62, 3.38] | Low^#%^ |
| Quetiapine vs Cariprazine | 0 |  | |  | 1.33 [0.58, 2.66] | Low^#%^ |
| Cariprazine vs placebo | 3 | 2.11 [1.48, 3.00] | | Moderate^ | 2.26 [1.26, 3.83] | Moderate^ |
| Cariprazine vs Aripiprazole | 0 |  | |  | 1.28 [0.49, 2.77] | Low^#%^ |
| Aripiprazole vs placebo | 2 | 1.85 [1.10, 3.10] | | Low*^ | 1.98 [0.96, 3.64] | Very low*^%^^ |
| **Odds Ratios for Akathisia** | | | | | | |
| Lurasidone vs placebo | 1 | 4.20 [1.90, 9.31] | | High | 9.50 [0.67,36.33] | High |
| Lurasidone vs Aripiprazole | 0 |  | |  | 1.45 [0.04,6.36] | Low^#%^ |
| Lurasidone vs Cariprazine | 0 |  | |  | 6.61 [0.15,13.84] | Low^#%^ |
| Cariprazine vs placebo | 4 | 3.10 [1.90, 5.06] | | High | 3.79 [1.19,9.27] | High |
| Cariprazine vs Aripiprazole | 0 |  | |  | 0.57 [0.06,2.23] | Low^#%^ |
| Aripiprazole vs placebo | 2 | 8.21 [4.56, 14.79] | | Moderate* | 12.15 [2.07,39.95] | Moderate* |
| **Odds Ratios for Switch to Mania** | | | | | | |
| Lurasidone vs placebo | 1 | 1.53 [0.53, 4.40] | | Moderate^%^ | 2.36 [0.39, 8.83] | Moderate^%^ |
| Lurasidone vs Aripiprazole | 0 |  | |  | 1.31 [0.14, 5.39] | Low^#%^ |
| Lurasidone vs Cariprazine | 0 |  | |  | 2.96 [0.39, 11.82] | Low^#%^ |
| Lurasidone vs Olanzapine | 0 |  | |  | 3.74 [0.43, 15.93] | Low^#%^ |
| Lurasidone vs Quetiapine | 0 |  | |  | 4.17 [0.61, 16.11] | Low^#%^ |
| Quetiapine vs placebo | 6 | 0.57 [0.34, 0.93] | | High | 0.61 [0.35, 0.99] | High |
| Quetiapine vs Aripiprazole | 0 |  | |  | 0.34 [0.10, 0.83] | Moderate^#^ |
| Quetiapine vs Cariprazine | 0 |  | |  | 0.77 [0.29, 1.69] | Low^#%^ |
| Quetiapine vs Olanzapine | 0 |  | |  | 0.95 [0.30, 2.46] | Low^#%^ |
| Olanzapine vs placebo | 3 | 0.79 [0.43, 1.44] | | Moderate^%^ | 0.79 [0.28, 1.67] | Low^#%^ |
| Olanzapine vs Aripiprazole | 0 |  | |  | 0.44 [0.09, 1.22] | Low^#%^ |
| Olanzapine vs Cariprazine | 0 |  | |  | 0.99 [0.26, 2.58] | Low^#%^ |
| Cariprazine vs placebo | 4 | 0.91 [0.56, 1.47] | | Moderate^%^ | 0.91 [0.41, 1.71] | Moderate^%^ |
| Cariprazine vs Aripiprazole | 0 |  | |  | 0.50 [0.13, 1.33] | Low^#%^ |
| Aripiprazole vs placebo | 2 | 1.96 [0.95, 4.03] | | Low*^%^ | 2.25 [0.81, 5.19] | Low*^%^ |

Factors downgrading any specific evidence: * Limitations (risk of bias), $ Inconsistency of results, # indirectness of results, % Imprecision, ^ Publications bias

**Appendix Table 8.** **Heterogeneity Assessment through Tau^2^ of the networks**

| **Outcome** | **Between study variance Tau^2^** |
| --- | --- |
| Change from Baseline in MADRS | 0.24 |
| Change from Baseline in CGI-BP-S Overall | 0 |
| Change from Baseline in CGI-BP-S Depression | 0.01 |
| Odds Ratio for Response | 0 |
| Odds Ratios for Remission (MADRS≤ 12) | 0 |
| Odds Ratios for Remission (MADRS≤ 10) | 0.02 |
| Odds Ratios for All-Cause Discontinuation | 0.03 |
| Odds Ratios for Discontinuation Due to Adverse Events | 0.11 |
| Odds Ratios for Discontinuation Due Lack of Efficacy | 0.26 |
| Change from Baseline in Weight | 0.05 |
| Odds Ratios of ≥7% Weight Gain | 0.09 |
| Change from Baseline in Triglycerides | 2.86 |
| Change from Baseline in Total Cholesterol | 12.57 |
| Change from Baseline in Low-Density Lipoprotein Cholesterol | 0.3 |
| Change from Baseline in Blood Glucose | 0.92 |
| Change from Baseline in Prolactin | 0.17 |
| Odds Ratios for Somnolence | 0.01 |
| Odds Ratios for Extrapyramidal Symptoms | 0.05 |
| Odds Ratios for Akathisia | 0.69 |
| Odds Ratio for Switch to Mania | 0.09 |

**Appendix Figure 1a.** **Network Diagrams for Change from Baseline in MADRS, CGI-BP-S Overall, CGI-BP-S-Depression, Weight and Blood Glucose**


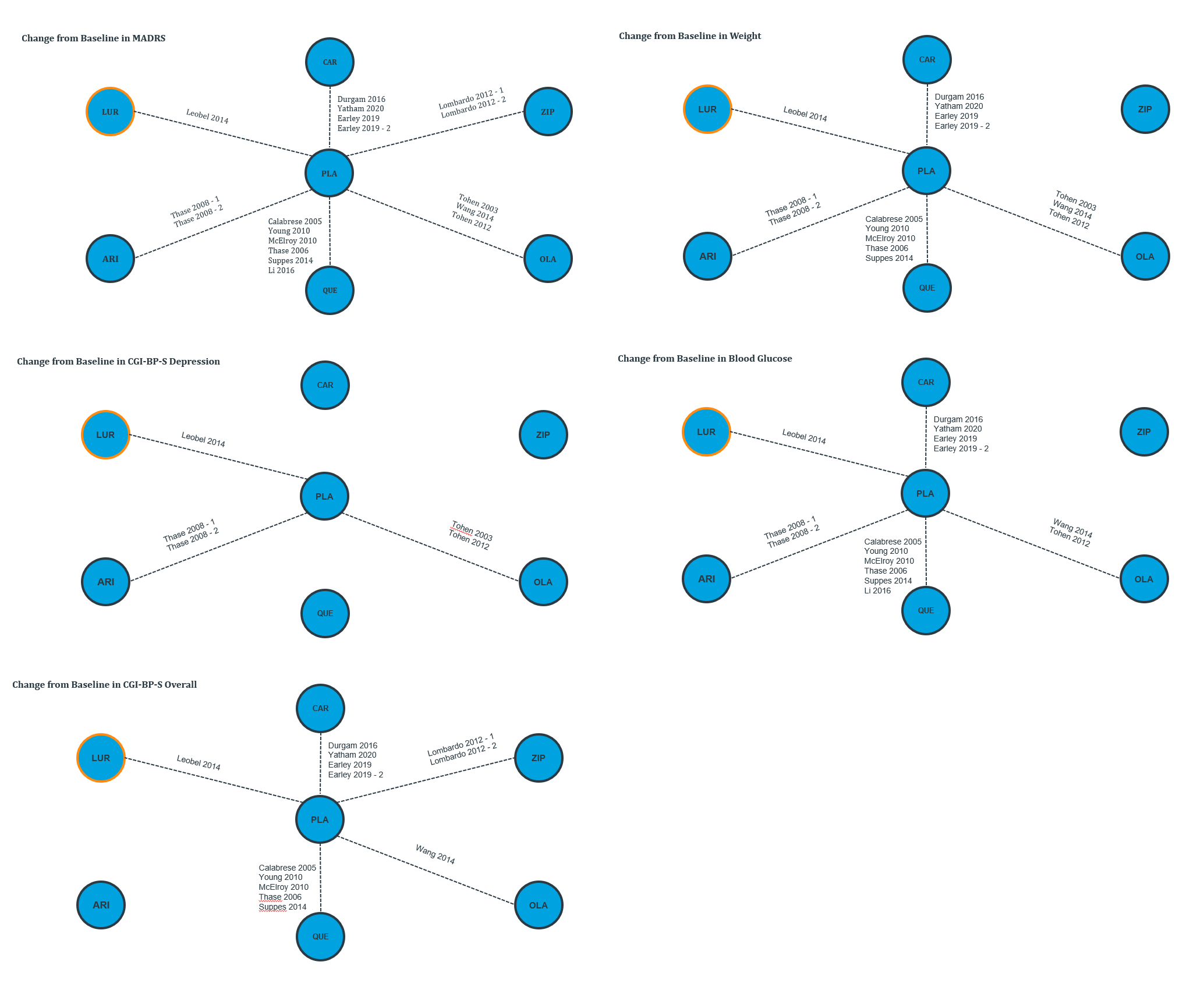


Abbreviations: PLO – Placebo; ARI - Aripiprazole; CAR – Cariprazine; LUR – Lurasidone; OLA – Olanzapine; QUE – Quetiapine; ZIP – Ziprasidone

**Appendix Figure 1b. Network Diagrams for Change from Baseline in Triglycerides, Total Cholesterol, Low-Density Lipoprotein Cholesterol and Prolactin**
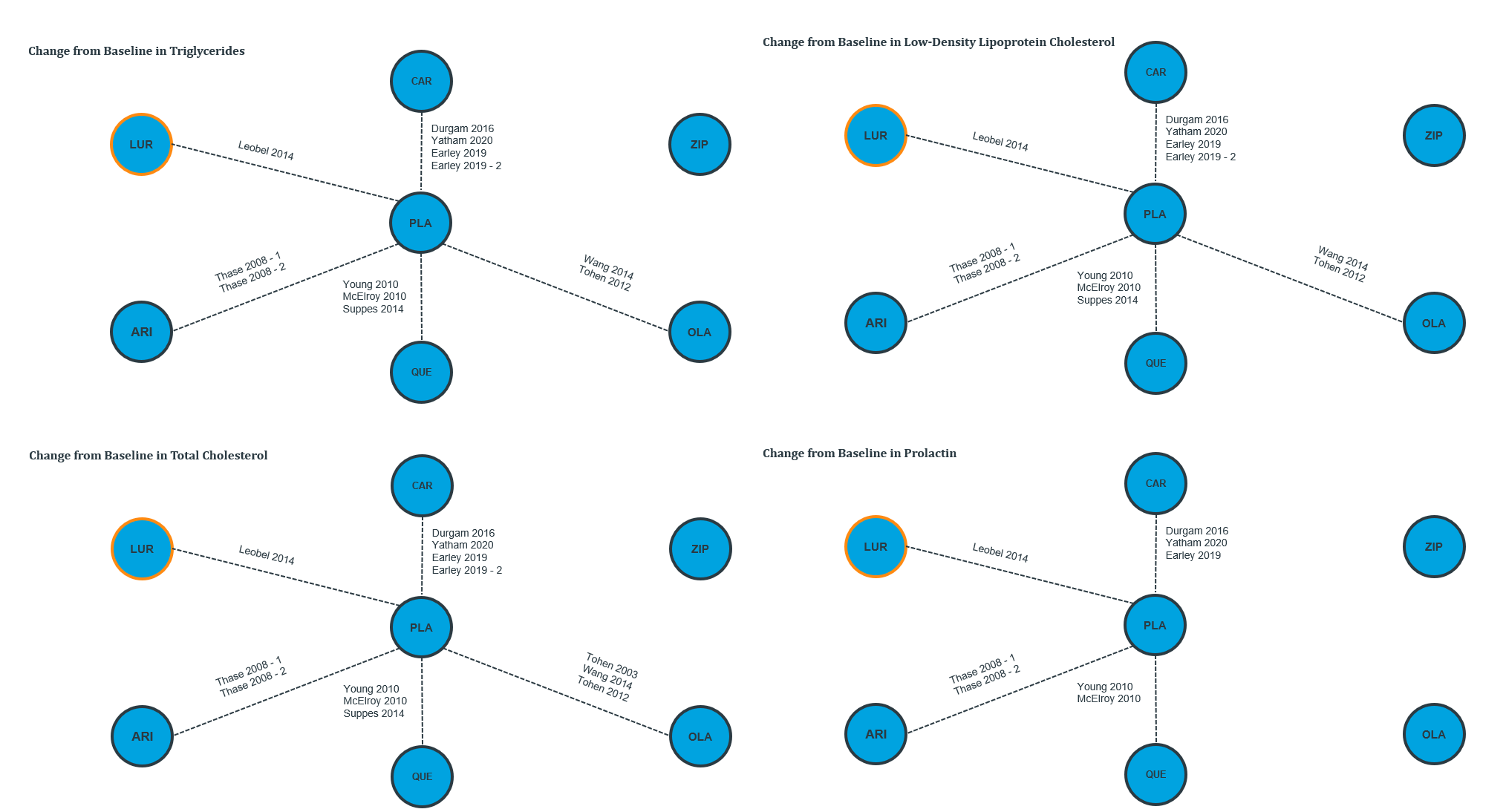


Abbreviations: PLO – Placebo; ARI - Aripiprazole; CAR – Cariprazine; LUR – Lurasidone; OLA – Olanzapine; QUE – Quetiapine; ZIP – Ziprasidone

**Appendix Figure 1c. Network Diagrams for Response, Remission (MADRS≤ 12 and ≤ 10), All Cause Discontinuation, Discontinuation due to Lack of Efficacy and Discontinuation due to Adverse Events**
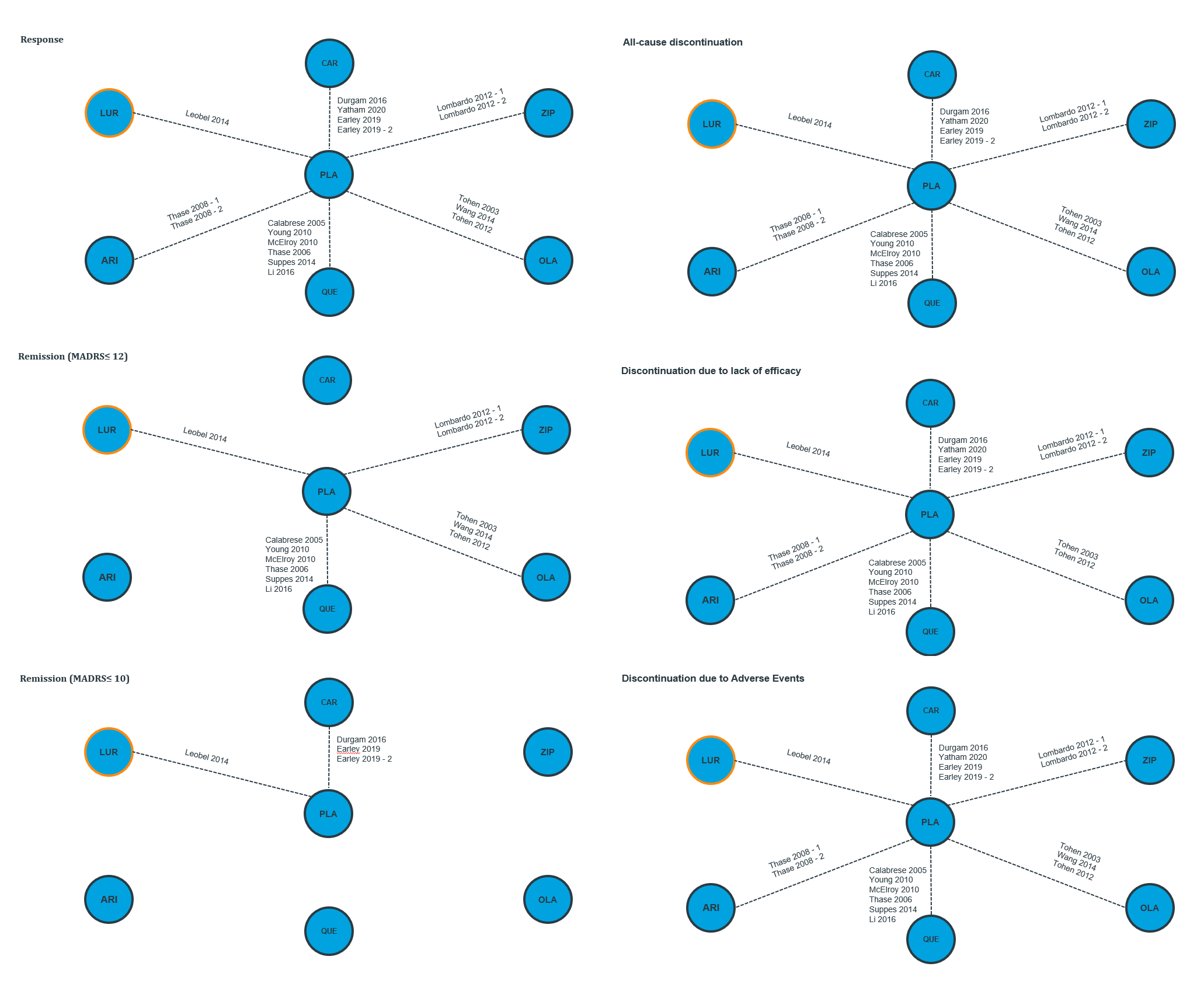


Abbreviations: PLO – Placebo; ARI - Aripiprazole; CAR – Cariprazine; LUR – Lurasidone; OLA – Olanzapine; QUE – Quetiapine; ZIP – Ziprasidone

**Appendix Figure 1d. Network Diagrams for ≥7% weight gain, Akathisia, Switch to Mania, Extrapyramidal Symptoms and Somnolence**
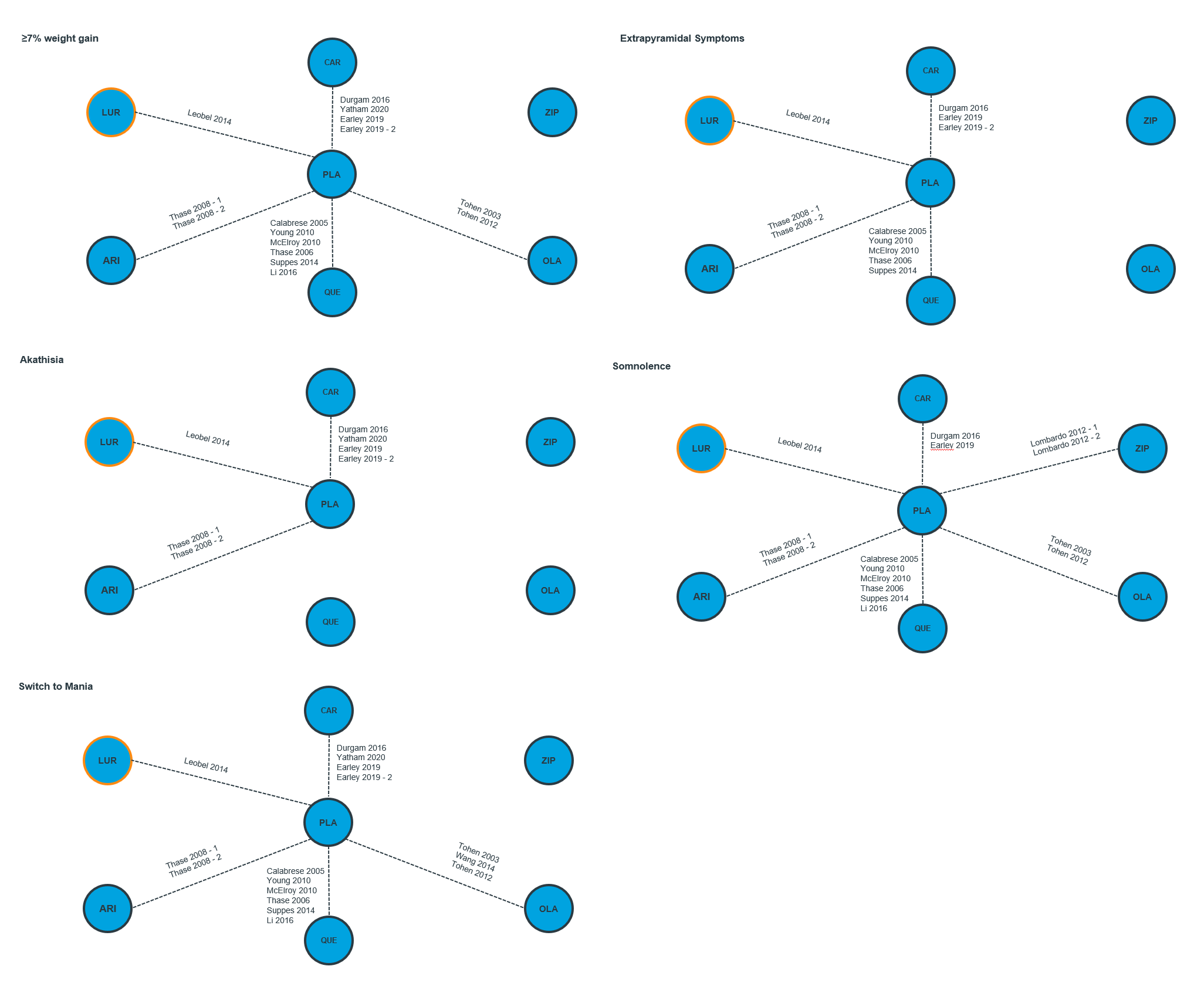


Abbreviations: PLO – Placebo; ARI - Aripiprazole; CAR – Cariprazine; LUR – Lurasidone; OLA – Olanzapine; QUE – Quetiapine; ZIP – Ziprasidone

**Appendix Figure 2.** **Risk of Bias Assessment of Included Studies**


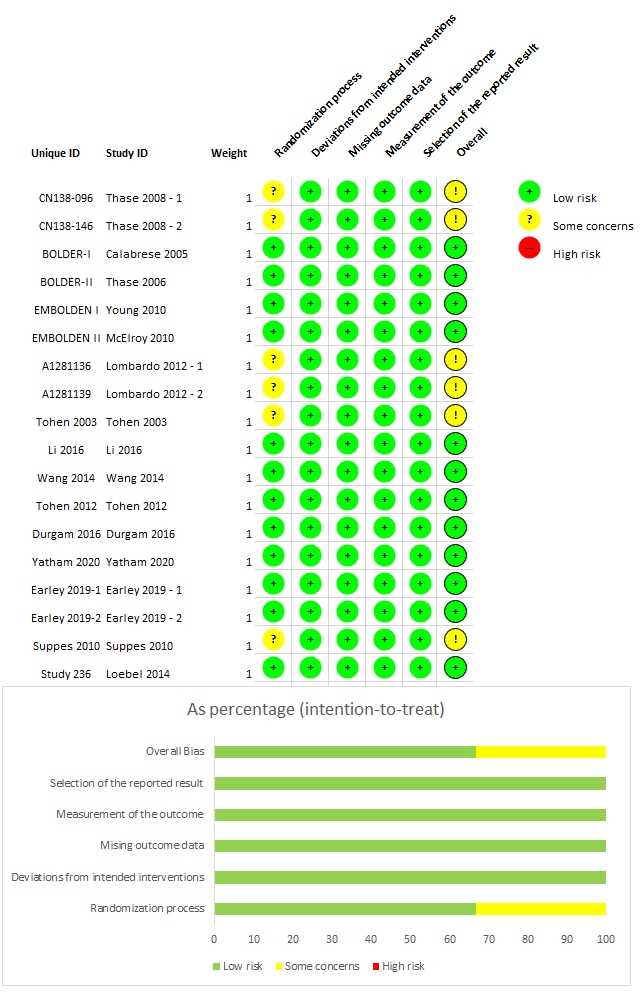


**Appendix Figure 3a.** **Assessment of Publication Bias Through Funnel Plots for Change from Baseline in MADRS, CGI-BP-S Overall, CGI-BP-S-Depression, Weight and Blood Glucose**


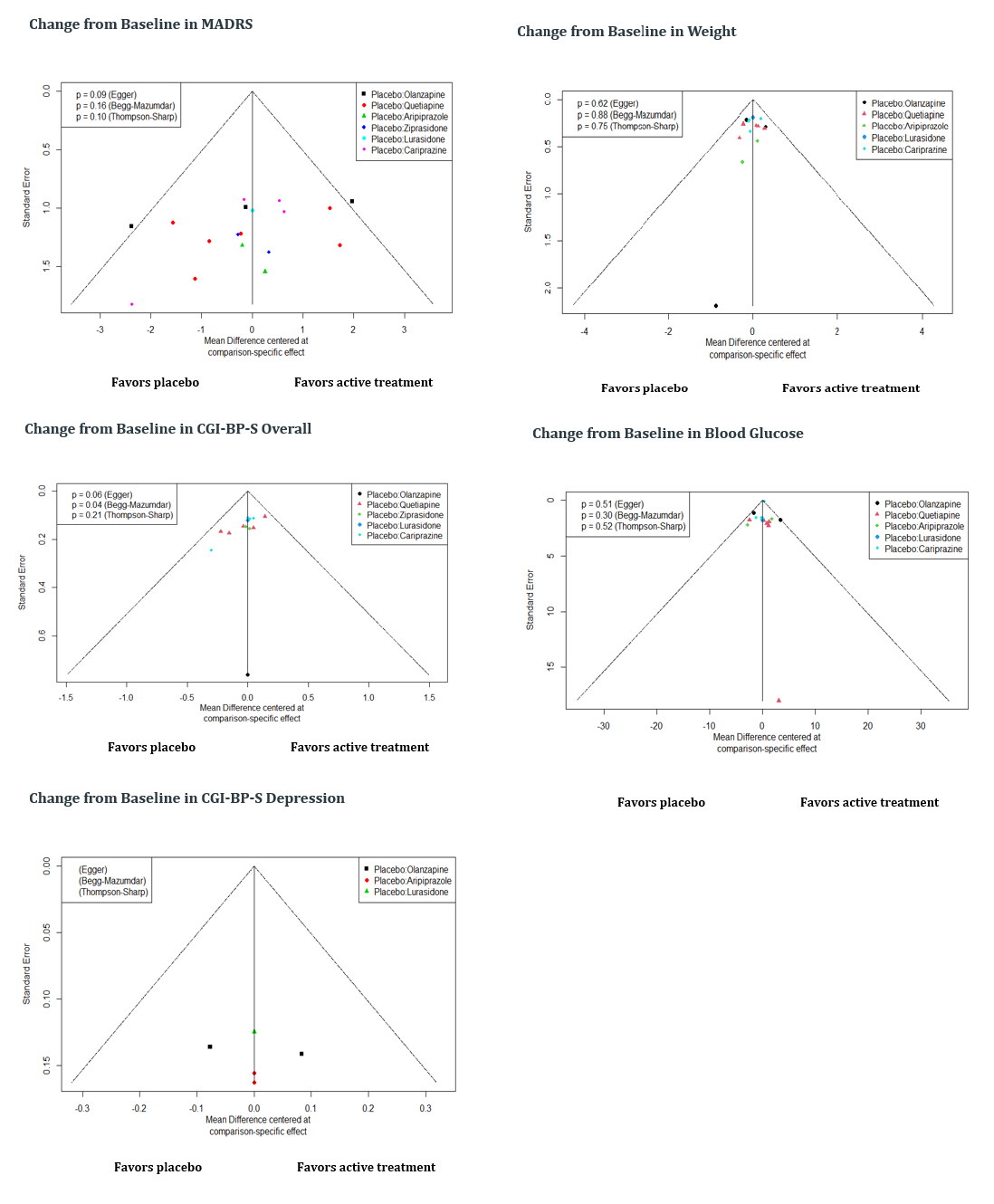


**Appendix Figure 3b. Assessment of Publication Bias Through Funnel Plots for Triglycerides, Total Cholesterol, Low-Density Lipoprotein Cholesterol and Prolactin**


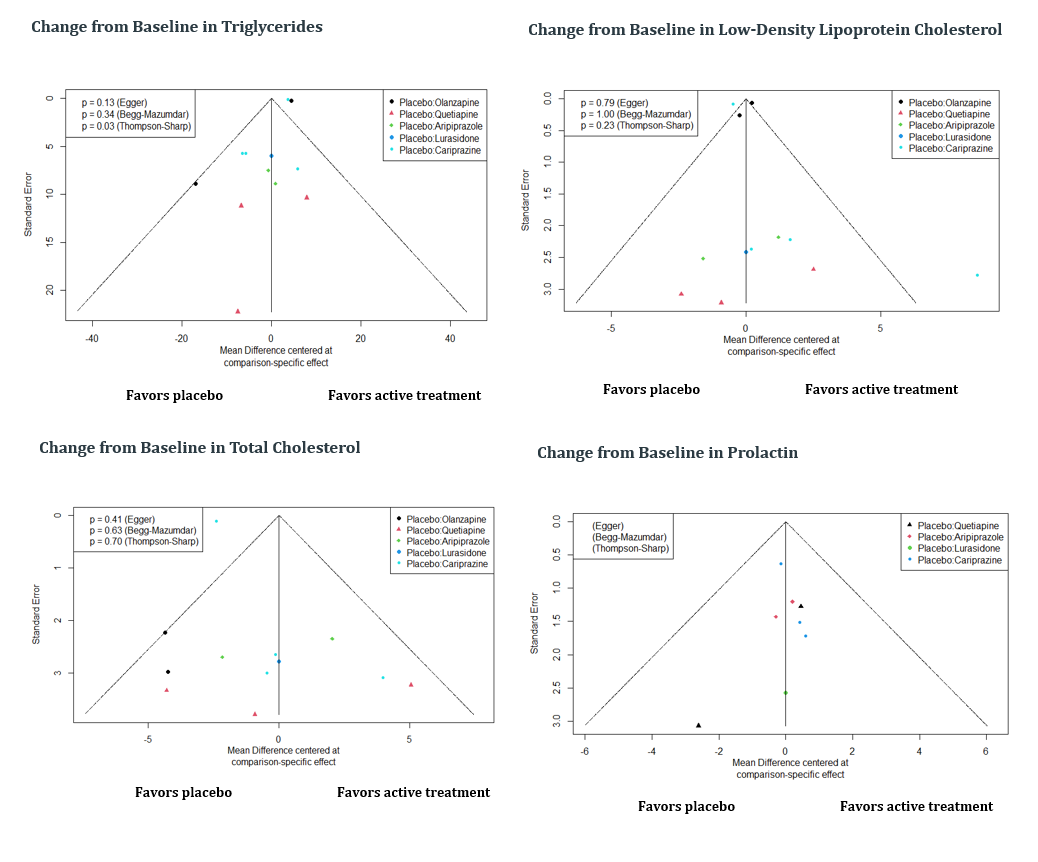


**Appendix Figure 3c. Assessment of Publication Bias Through Funnel Plots for Response, Remission (MADRS≤ 12 and ≤ 10), All Cause Discontinuation, Discontinuation due to Lack of Efficacy and Discontinuation due to Adverse Events**


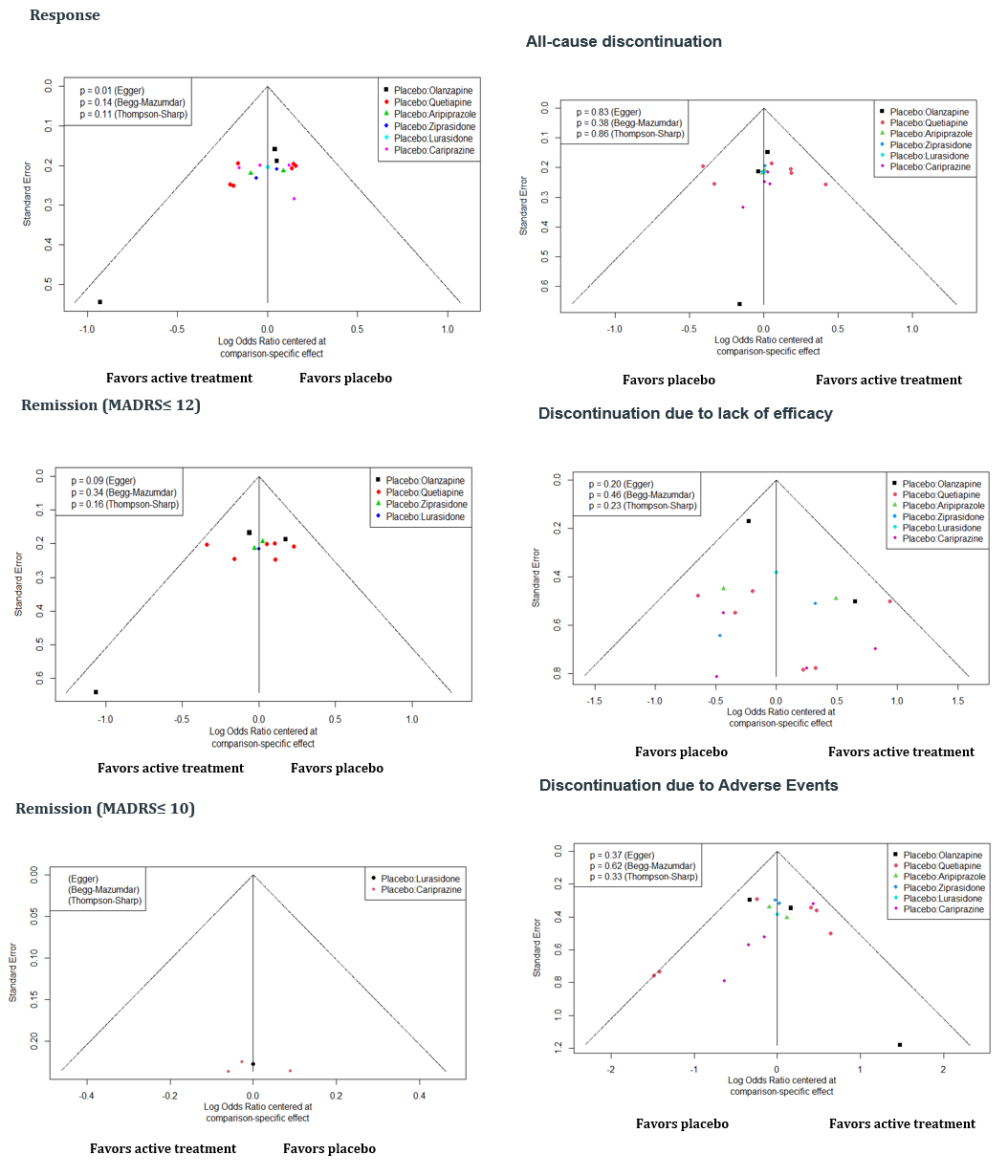


**Appendix Figure 3d.** **Assessment of Publication Bias Through Funnel Plots for ≥7% weight gain, Akathisia, Switch to Mania, Extrapyramidal Symptoms and Somnolence**


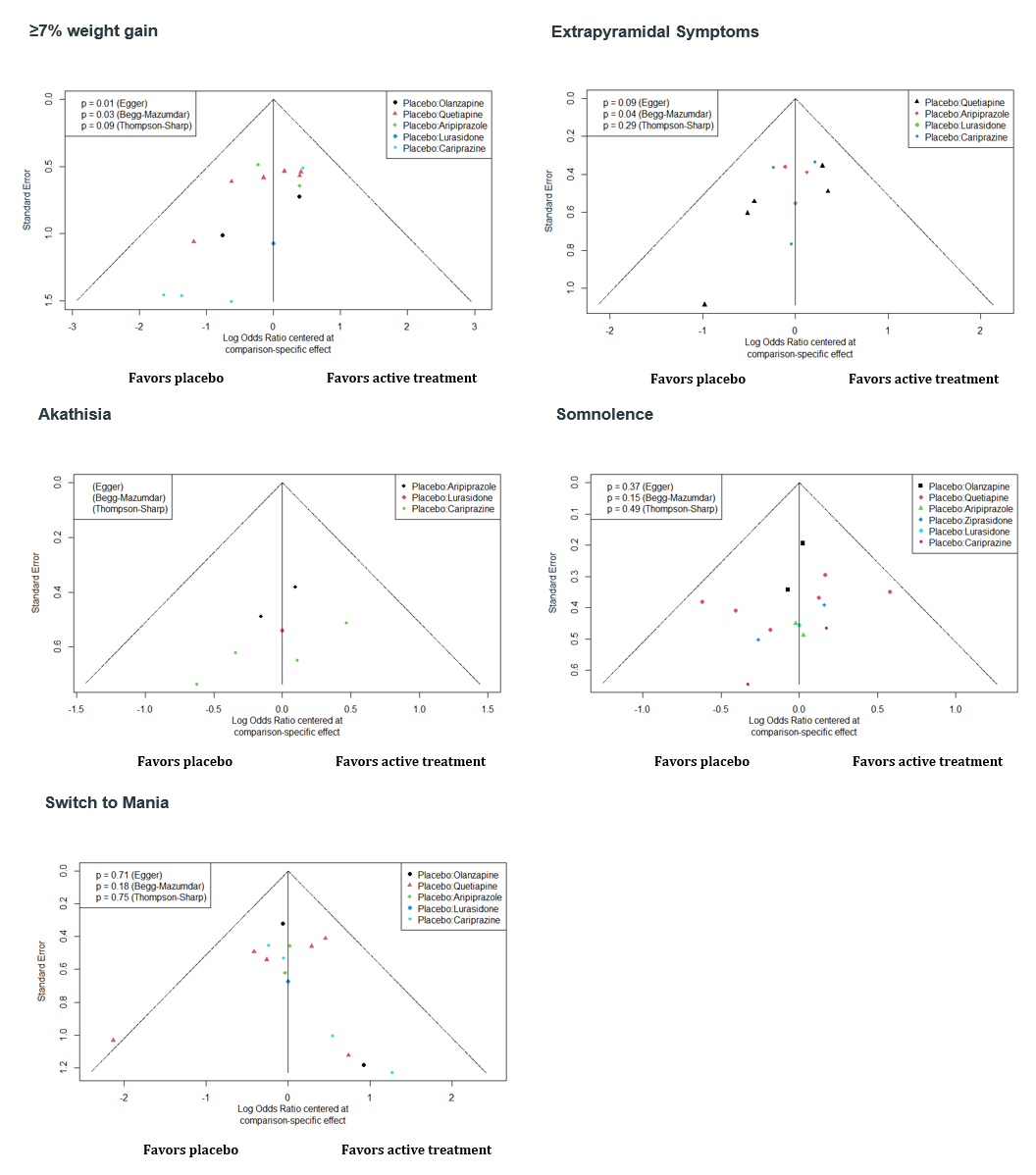


**Appendix Figure 4a.** **Heterogeneity Assessment of Included RCTs Through Effect Modifier Comparison**


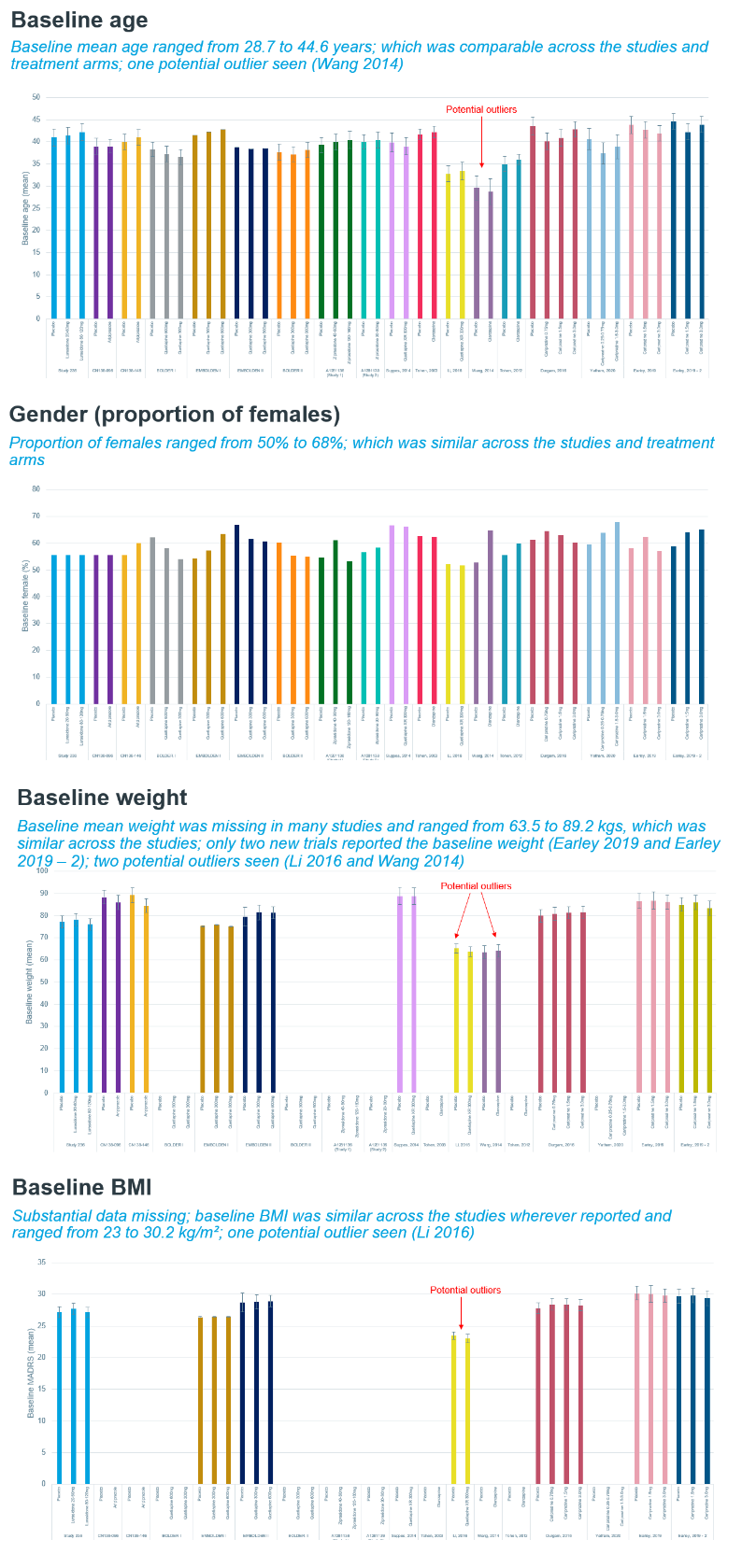


Abbreviations: PLO – Placebo; ARI - Aripiprazole; CAR – Cariprazine; LUR – Lurasidone; OLA – Olanzapine; QUE – Quetiapine; ZIP – Ziprasidone

**Appendix Figure 4b. Heterogeneity Assessment of Included RCTs Through Effect Modifier Comparison (Contd.)**


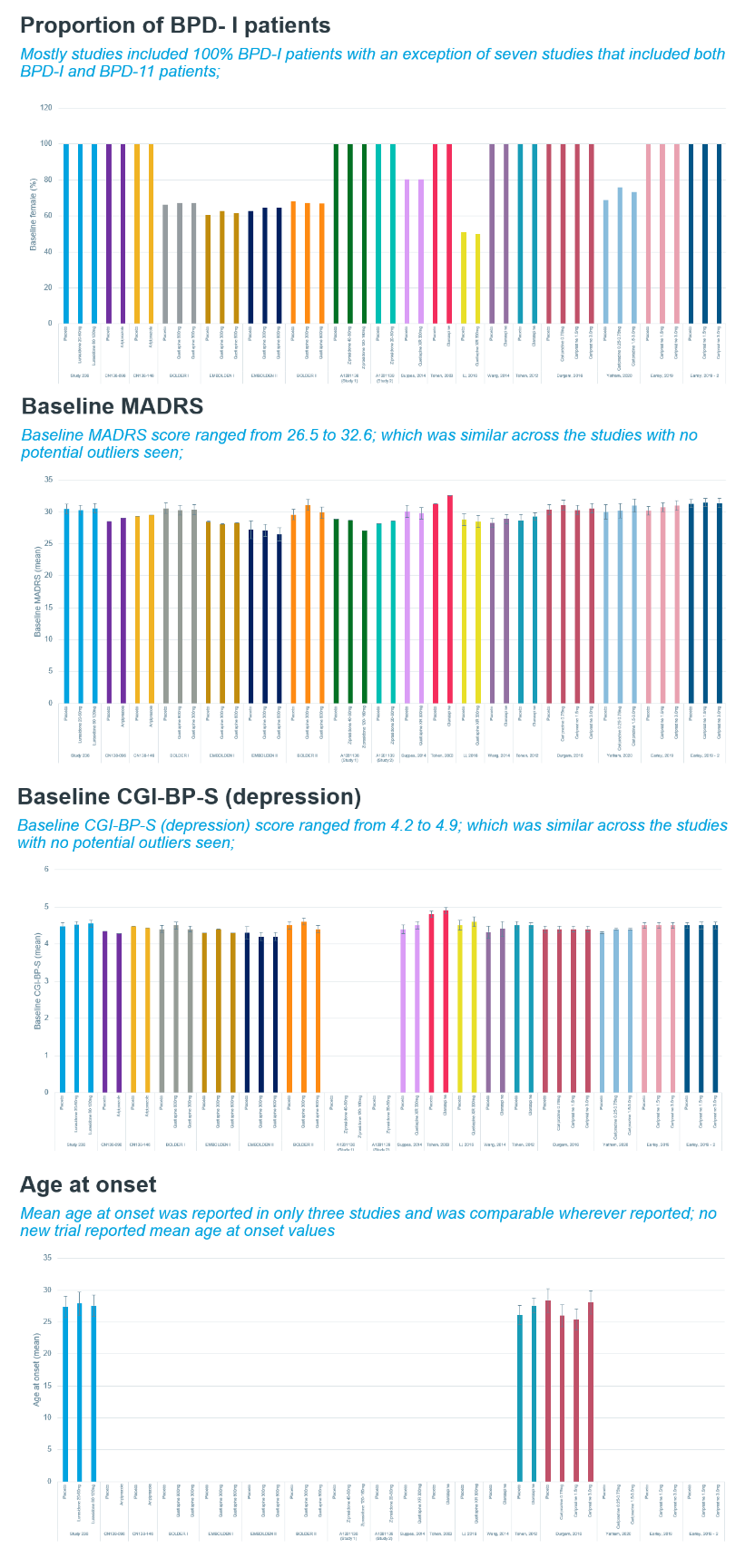


Abbreviations: PLO – Placebo; ARI - Aripiprazole; CAR – Cariprazine; LUR – Lurasidone; OLA – Olanzapine; QUE – Quetiapine; ZIP – Ziprasidone
